# Macrophage infection, activation, and histopathological findings in ebolavirus infection

**DOI:** 10.3389/fcimb.2022.1023557

**Published:** 2022-10-12

**Authors:** Timothy G. Wanninger, Daniel E. Millian, Omar A. Saldarriaga, Junki Maruyama, Takeshi Saito, Rachel A. Reyna, Satoshi Taniguchi, Esteban Arroyave, Melanie E. Connolly, Heather L. Stevenson, Slobodan Paessler

**Affiliations:** ^1^ Department of Pathology, University of Texas Medical Branch, Galveston, TX, United States; ^2^ Department of Microbiology and Immunology, University of Texas Medical Branch, Galveston, TX, United States; ^3^ Department of Surgery, University of Texas Medical Branch, Galveston, TX, United States

**Keywords:** filovirus, pathology, histology, immunity, macaque, liver, human

## Abstract

Macrophages contribute to Ebola virus disease through their susceptibility to direct infection, their multi-faceted response to ebolaviruses, and their association with pathological findings in tissues throughout the body. Viral attachment and entry factors, as well as the more recently described influence of cell polarization, shape macrophage susceptibility to direct infection. Moreover, the study of Toll-like receptor 4 and the RIG-I-like receptor pathway in the macrophage response to ebolaviruses highlight important immune signaling pathways contributing to the breadth of macrophage responses. Lastly, the deep histopathological catalogue of macrophage involvement across numerous tissues during infection has been enriched by descriptions of tissues involved in sequelae following acute infection, including: the eye, joints, and the nervous system. Building upon this knowledge base, future opportunities include characterization of macrophage phenotypes beneficial or deleterious to survival, delineation of the specific roles macrophages play in pathological lesion development in affected tissues, and the creation of macrophage-specific therapeutics enhancing the beneficial activities and reducing the deleterious contributions of macrophages to the outcome of Ebola virus disease.

## Introduction

Ebolavirus infection is a threat to human health in both Sub-Saharan Africa and abroad. The first recorded ebolavirus outbreak occurred in 1976 in the Democratic Republic of the Congo, formerly known as Zaire, with subsequent outbreaks occurring throughout the Sub-Saharan region, from Sierra Leone in the west, to Uganda in the east (Malvy et al., 2019). The asymptomatic infection of an animal handler in Virginia, United States of America, has also been documented (Albariño et al., 2017). The largest outbreak to date occurred from 2013-2016 in the countries of Guinea, Sierra Leone, and Liberia, with over 28,000 cases, including cases exported to other African countries, Europe, and the United States (Malvy et al., 2019; Centers for Disease Control and Prevention, 2022; World Health Organization, 2022). As recently as 2022, an outbreak occurred in the Democratic Republic of the Congo (World Health Organization, 2022). Since 1976, human cases of infection have been reported for five of the six known species in the genus *Ebolavirus*: *Zaire ebolavirus*, *Sudan ebolavirus*, *Reston ebolavirus*, *Tai Forest ebolavirus*, and *Bundibugyo ebolavirus*, *Bombali ebolavirus* being the exception. Representative viruses are Ebola virus (EBOV), Sudan virus (SUDV), Reston virus (RESTV), Taï Forest virus (TAFV), Bundibugyo virus (BDBV), and Bombali virus (BOMV), respectively. The fatality rate averages to roughly 45% across all known human outbreaks, with fatality rates ranging from 0%, in the case of RESTV, to greater than 80% in some EBOV outbreaks (Kuhn et al., 2021). Natural infection of primates with ebolaviruses has also been documented (Geisbert et al., 1992; Wyers et al., 1999).

Like other filoviruses, ebolaviruses are filamentous negative-sense, single-stranded ribonucleic acid (RNA) viruses (Rougeron et al., 2015). The RNA genome encodes for seven viral proteins: nucleoprotein (NP), polymerase cofactor (VP35), matrix protein (VP40), glycoprotein (GP), transcriptional activator (VP30), RNA complex-associated protein (VP24), and large protein (L). NP, VP24, VP30, VP35, and the L polymerase manage replication of the RNA genome. VP40 mediates recruitment of the resulting ribonucleoprotein complexes to the cell membrane. GP is a type I transmembrane protein that facilitates virus attachment and entry. Six products of the *GP* gene have been reported: GP, shed GP, secreted GP (sGP), sGP-GP subunit 2 heterodimers, secondary secreted GP, and Δ-peptide (Kuhn et al., 2021). In addition to these roles, several of these viral proteins, including VP24, VP30, VP35, VP40, GP, shed GP, and sGP regulate the immune response to ebolavirus infection (Baseler et al., 2017; Kuhn et al., 2021).

Ebolavirus infection is not only frequently fatal in humans, but it also can cause chronic sequelae. Direct contact with body fluids, which can be contaminated with the virus, is the most likely form of transmission. EBOV isolation (blood, breast milk, saliva, semen) or RNA detection (rectal swab, vaginal swab, skin, stool, sweat, tears) has been done for various specimens, indicating multiple avenues for potential exposure to infectious virus (Judson et al., 2015). Early symptoms of disease include fever, fatigue, anorexia, myalgia, and headache, followed by gastrointestinal symptoms, such as nausea, vomiting, and diarrhea. In severe cases, patients can develop multi-organ dysfunction syndrome. Following the resolution of acute infection, patients can experience fatigue, uveitis, hearing loss, arthralgia, insomnia, and other sequelae (Jacob et al., 2020).

Macrophages are one of the key cells implicated in the pathogenesis of Ebola virus disease (EVD). Infection of macrophages, as well as dendritic cells, is thought to occur initially in dermal or submucosal tissues, or within lymph nodes. The infection may then progress systemically to other tissues through the circulation (Schnittler and Feldmann, 1998; Kuhn et al., 2021). Certain organs, such as the liver and spleen, may be more rapidly susceptible to the virus as their resident macrophage populations interact directly with the blood (Schnittler and Feldmann, 1998). Macrophages respond to the infection with dysregulated cytokine production, in contrast to dendritic cells, which demonstrate impaired activation following infection (Mahanty et al., 2003; Leung et al., 2011; Rogers and Maury, 2018). The various subpopulations of macrophages, distinguished both by lineage (resident vs monocyte-derived) and polarization, including M1 (pro-inflammatory Th1 response) and M2 (anti-inflammatory, Th2 activation, phagocytosis of apoptotic cells, angiogenesis, depending on the phenotype) macrophages, lay the foundation for a complex response by this cell population (Shapouri-Moghaddam et al., 2018). Much research, summarized in this review, has focused on understanding (macrophage), susceptibility to ebolavirus infection, macrophage activation, and pathological findings associated with macrophages in EVD.

## Ebolavirus entry into macrophages

**Figure 1:**
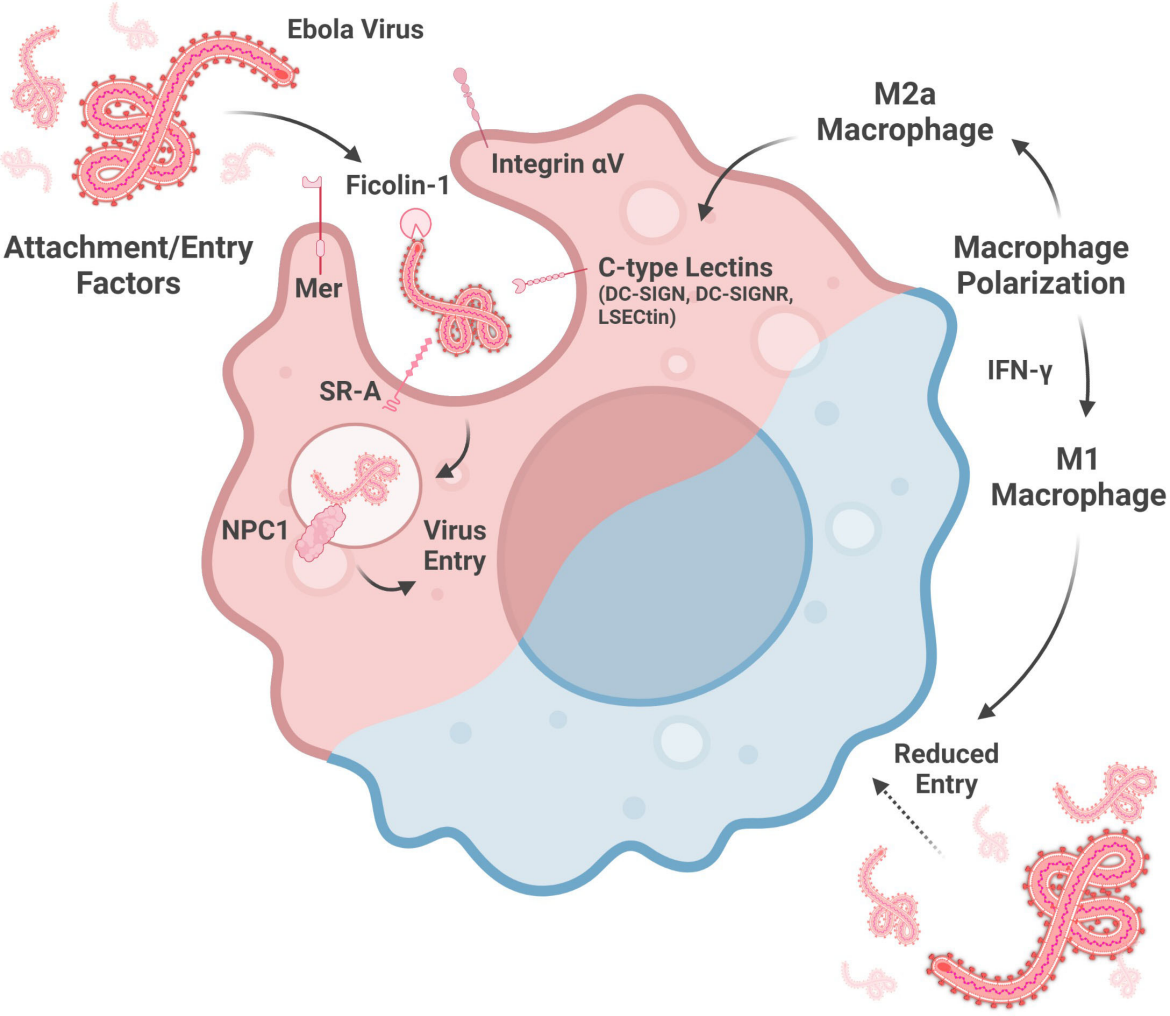
Factors altering macrophage susceptibility to ebolavirus entry. Entry into the cytoplasm is mediated by attachment factors, which facilitate virion adherence to the cell surface, and entry factors, which are involved in membrane fusion. Macrophage susceptibility is enhanced by a M2a phenotype, associated with increased C-type lectin expression, and reduced by a M1 phenotype, driven by IFN-y signaling. (Created with Biorender.com).

Multiple C-type lectins enhance ebolavirus entry into host cells by mediating virus attachment to macrophages (**Figure 1**). Macrophages transduced with lentiviruses encoding either Dendritic Cell-specific Intercellular Adhesion Molecule 3-grabbing Non-integrin (DC-SIGN), one of the titular members of this sugar-binding protein family, or DC-SIGN-related protein (DC-SIGNR) show enhanced EBOV entry, though the expression level of these lectins in this model needs to be confirmed (Simmons et al., 2003; Zhang et al., 2014). However, the inability to make T cells susceptible to EBOV GP-pseudotyped Human Immunodeficiency virus infection by DC-SIGN- or DC-SIGNR-encoding lentivirus transduction indicates that, while these lectins may facilitate virion binding, they are not necessarily sufficient to facilitate virus entry (Simmons et al., 2003). Nevertheless, as DC-SIGN is expressed by alveolar macrophages in the lung and decidual macrophages in the placenta, DC-SIGN, and its relatives, are important to consider for understanding macrophage susceptibility to ebolavirus infection (Soilleux et al., 2002).

Indeed, DC-SIGN and DC-SIGNR are not the only C-type lectins implicated in macrophage susceptibility to ebolavirus infection. Liver and lymph node sinusoidal endothelial cell C-type lectin (LSECtin) is expressed by Kupffer cells, the resident phagocytes of the liver, and by thymic macrophages (Dominguez-Soto et al., 2007; Domínguez-Soto et al., 2009). This protein may also serve as an ebolavirus attachment factor as EBOV GP binding to dendritic cells is partially abrogated by anti-LSECtin polyclonal antiserum and because transfection-induced LSECtin expression in a chronic myelogenous leukemia cell line enhanced EBOV GP-pseudotyped lentivirus entry (Dominguez-Soto et al., 2007).

In addition to the C-type lectins, several other proteins contribute to ebolavirus attachment to, or entry into, macrophages (**Figure 1**). siRNA knock-down of Niemann-Pick C1 (NPC1), Mer, Integrin αV, or Scavenger Receptor A (SR-A) reduces the entry of lentivirus expressing EBOV GP into macrophages, as measured at 72 hours post-infection. SR-A inhibition by tannic acid pre-incubation also reduces entry of this pseudotyped virus into macrophages in a dose-dependent manner. In contrast, EBOV infection of macrophages was reduced by siRNA knock-down of Integrin αV or a combination knock-down of Mer, SR-A, and NPC1, but not Mer, SR-A, nor NPC1 alone (Dahlmann et al., 2015). NPC1-mediated entry is influenced by the GP sequence, as the F88A mutation in GP is associated with reduced entry into macrophages. GP interaction with human NPC1 appears to be more sensitive to this mutation than murine NPC1 (Martinez et al., 2013b). Lastly, Ficolin-1, a defense collagen protein activating the lectin complement pathway, both binds to EBOV GP and also enhances EBOV infection of macrophages (Favier et al., 2016; Casals et al., 2019). Taken together, NPC1, a protein well-known for its role in EBOV entry, as well as other cellular proteins, contribute to ebolavirus entry into macrophages. Future study of the multiple ebolavirus attachment and entry factors will benefit from use of true virus as much work to date has been done using pseudotyped viruses, which may not completely replicate the function of actual virions.

Research has also been done on whether monocytes, which can differentiate into macrophages, can also be infected by ebolaviruses. Monocytes have been reported to support ebolavirus replication when looking at one or more days post-infection. These cells even produced TNF-α, IL-6, IL-8, and gro-alpha in response to EBOV and RESTV, which were also produced by macrophages infected with the same viruses (Ströher et al., 2001). Further work in this area has shown that monocytes begin to support EBOV virus-like particle (VLP) entry after 20 hours in culture. THP-1 cells (human monocyte-like cell line) treated with phorbol myristate acetate (PMA) to induce differentiation showed a trend toward enhanced EBOV VLP entry. However, treating monocytes with PMA showed a trend toward reduced EBOV VLP entry (Martinez et al., 2013a). Thus, while cell differentiation status may influence susceptibility to ebolavirus infection, this phenomenon remains to be fully clarified in monocytes. Further study of ebolavirus infection of monocytes will benefit from the use of real virus as actual virions may act in a way incompletely recapitulated by VLPs.

Recent work has shown that macrophage polarization is also key to susceptibility to ebolavirus infection (**Figure 1**). Polarization towards a M2a phenotype (anti-inflammatory, tissue remodeling phenotype) is associated with enhanced EBOV GP-pseudotyped vesicular stomatitis virus (VSV) infection in human monocyte-derived macrophages and murine peritoneal macrophages as well as increased expression of the C-type lectins SIGNR3 and SIGNR5 in murine peritoneal macrophages (Shapouri-Moghaddam et al., 2018; Rogers et al., 2019). LSECtin, one of the proposed ebolavirus attachment factors expressed on Kupffer cells, is induced by IL-4 stimulation, consistent with alternative activation (Dominguez-Soto et al., 2007). Moreover, IL-10, which is associated with the induction of a M2c phenotype (associated with phagocytosis of apoptotic cells), enhances EBOV VLP entry into human monocyte-derived macrophages (Shapouri-Moghaddam et al., 2018; Stantchev et al., 2019). In contrast, macrophage polarization towards a M1 phenotype may protect macrophages from infection. CD40 has been shown to be important for IL-12-mediated IFN-γ production (IFN-γ contributes to a M1 phenotype), which reduces EBOV infection of murine peritoneal macrophages (Rogers et al., 2021; Shapouri-Moghaddam et al., 2018). In addition, serum from *Plasmodium yoelii*-infected mice, which contains high levels of IFN-γ, also protects mouse peritoneal macrophages against EBOV GP-pseudotyped VSV infection (Rogers et al., 2020). Several interferon-stimulated genes upregulated by IFN-γ in human monocyte-derived macrophages, including Interferon regulatory factor 1, Vesicle-associated membrane protein 5, Guanylate Binding Protein 5, and Retinoic acid receptor responder protein 3, are associated with reduced EBOV infection of HeLa cells over-expressing these genes (Rhein et al., 2015). In summary, M1 macrophage polarization may be a protective response while M2 polarization may increase macrophage susceptibility to virus entry. Further studies on the role of macrophage polarization in ebolavirus entry with true virus and *in vivo* models will enrich this area of study by more closely replicating infection as compared to the pseudovirus or VLP models and *in vitro* macrophages used in several of these studies to date.

## Macrophage response to ebolavirus

**Figure 2:**
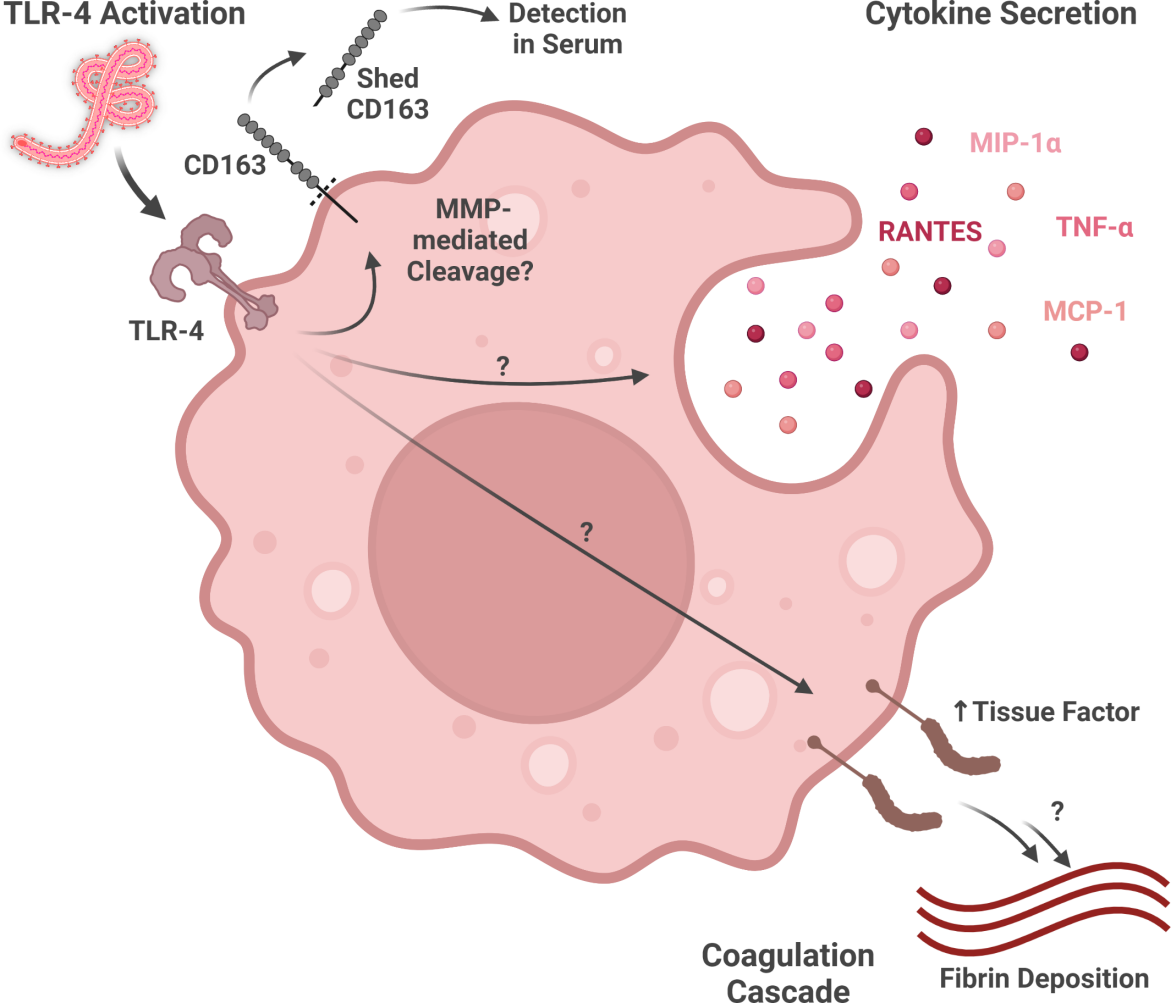
Macrophage activity during ebolavirus infection. Virion glycoprotein binding to TLR4 may activate multiple responses, including the shedding of CD163 (detectable in serum), the expression of various inflammatory cytokines and chemokines, and the expression of Tissue Factor (part of the coagulation cascade). (Created with Biorender.com).

Macrophages exposed to *Ebolavirus* species produce several different cytokines and chemokines (**Figure 2**). EBOV-infected human monocyte-derived macrophages produce TNF-α (proinflammatory cytokine), MIP-1α (chemokine attracting lymphocytes, neutrophils, and eosinophils), RANTES (chemokine attracting lymphocytes, neutrophils, and eosinophils), and MCP-1 (macrophage chemoattractant). Interestingly, these signaling molecules are even produced, to at least some extent, by irradiated virus, suggesting that mechanisms both dependent on, and independent of, virus replication contribute to this response (Gupta et al., 2001). Many of the gene expression changes induced by virions in primary human macrophages are also induced in a similar manner by GP-VP40 VLPs, but not VP40-only VLPs, suggesting that interactions between GP and macrophages are key for the activation profile of these cells (Wahl-Jensen et al., 2011).

More recent work has shed light on the role GP-activated Toll-like receptor signaling plays in macrophage activation by ebolaviruses (**Figure 2**). EBOV GP, but not RESTV GP, activates GP-mediated NF-kB signaling through Toll-like receptor 4 (TLR4) in macrophages (Olejnik et al., 2017). As many of the signaling molecules induced by EBOV-mediated macrophage activation, including TNF-α, MIP-1α, RANTES, and MCP-1, are expressed downstream of TLR4 signaling, this pathway may play a key role in the inflammatory response during EVD (Gupta et al., 2001; Newton and Dixit, 2012). Moreover, GP-mediated TLR4 signaling may help explain Tissue Factor, a downstream product of the NF-kB pathway involved in the coagulation cascade, expression by macrophages in *in vitro* and *in vivo* models of EBOV infection (Geisbert et al., 2003b; Newton and Dixit, 2012; Greenberg et al., 2020). As TLR4 antagonism has been shown to reduce murine mortality from ebolavirus infection, additional study of the contributions of TLR4 to severe and fatal EVD as well as TLR4-modulating therapeutics is warranted (Younan et al., 2017).

In addition to the production of inflammatory cytokines and chemokines, interferon production by macrophages also contributes to the immune response to *Ebolavirus* species. The activation of interferon correlates with ebolavirus pathogenicity, as EBOV promotes greater expression of Type-I and Type-III interferon genes, as well as interferon-stimulated genes, as compared to RESTV (Olejnik et al., 2017). EBOV VLPs also elicit early Type-I Interferon gene expression by macrophages (Ayithan et al., 2014). RIG-I-like receptor signaling has been studied in a loxP-flanked homologous recombination mitochondrial antiviral signaling protein knock-out (MAVS^-/-^) mouse model of EBOV infection, which has an impaired Type-I interferon response. Cell deconvolution of RNA expression in the spleens of these mice showed that the peak accumulation of macrophages in the spleen was delayed in the knockout mice compared with wildtype mice. Follow-up analysis of viral antigen in a monocyte/macrophage-specific MAVS knock-out mouse model (conditional cre-lox knock-out under the myeloid cell LysM promoter) showed that the lack of MAVS expression in these cells was associated with greater viral antigen in the liver and spleen as well as reduced mouse survival. Taken together, these data highlight that monocyte/macrophage RIG-I-like receptor signaling contributes to the antiviral response to EBOV (Dutta et al., 2017). These activations of the interferon pathway and their contributions to the inflammatory response are important to understand because EBOV antagonizes this response *via* VP24, which impairs STAT1 homodimer and STAT1/2 heterodimer signaling, and VP35, which interferes with multiple factors, including RIG-I, which is involved in the detection of viral RNA (Kühl and Pöhlmann, 2012).

CD163, a protein playing a role in other macrophage-related diseases, is also involved in EVD. Macrophages expressing CD163, a hemoglobin/haptoglobin scavenger receptor associated with anti-inflammatory functions, is present in multiple tissues, including the liver, spleen, heart, and testis (McElroy et al., 2019; Skytthe et al., 2020). Co-localization of EBOV antigen with CD163^+^ macrophages has been observed in the liver and spleen (McElroy et al., 2019). CD163 is shed from the cell by multiple mechanisms, including protease-mediated cleavage following TLR activation by lipopolysaccharide (**Figure 2**) (Hintz et al., 2002; Møller, 2012). While increases in soluble CD163 are associated with fatal ebolavirus infection in patients, this difference may be driven by a small number of outlier patients who have extremely high levels of this protein in their serum (McElroy et al., 2019). This marker also shows up in animal models of filovirus infection, with CD14^+^CD163^+^ macrophages (M2-like) showing a more terminally differentiated/exhausted phenotype in EBOV-challenged, compared to Marburg virus-challenged, Rag2^−/−^γc^−/−^CD47^−/−^ mice engrafted with fetal human bone marrow, liver, and thymus (Lavender et al., 2018). While CD163 has been highlighted as a potentially important marker in ebolavirus infection, further research defining its mechanistic or phenotypic significance to macrophage activity is needed.

Ebolavirus infection also induces changes at the gene expression level in monocyte/macrophage populations in the blood and in tissues. Single-cell RNA-seq analysis of peripheral blood mononuclear cells in a cynomolgus macaque (*Macaca fascicularis*) model of EBOV, using an algorithm to associate gene expression with cell type, predicted that many of the differentially expressed genes within the library were most likely expressed by monocyte-, macrophage- or dendritic-like cells (Versteeg et al., 2017). Microarray analysis of lung tissue from a porcine EBOV model highlighted increased expression of macrophage-related genes, including surface markers (CD14, CD163), cytokines/chemokines (IL-6, CCL2, AMCF-II), macrophage differentiation/activation-related proteins (GM-CSF, RETN), and TLRs (TLR4) (Nfon et al., 2013). Additionally, in a rhesus macaque (*Macaca mulatta*) BDBV model, myeloid-derived suppressor cell-related transcripts (*S100A8, S100A9, PTGS2, CEBPB, CXCR1, LILRA3*, and others), as well as *CD163*, were generally less elevated in macaques surviving mild-to-moderate disease versus those surviving severe disease, when normalized against fatal disease (Woolsey et al., 2021). Taken together, these data support macrophage activation status and signaling activity as noteworthy components of the immune response to ebolavirus infection as macrophage-related transcripts are frequently differentially expressed during ebolavirus infection.

## Macrophage histopathology in different organs during ebolavirus disease

### Liver

**Figure 3:**
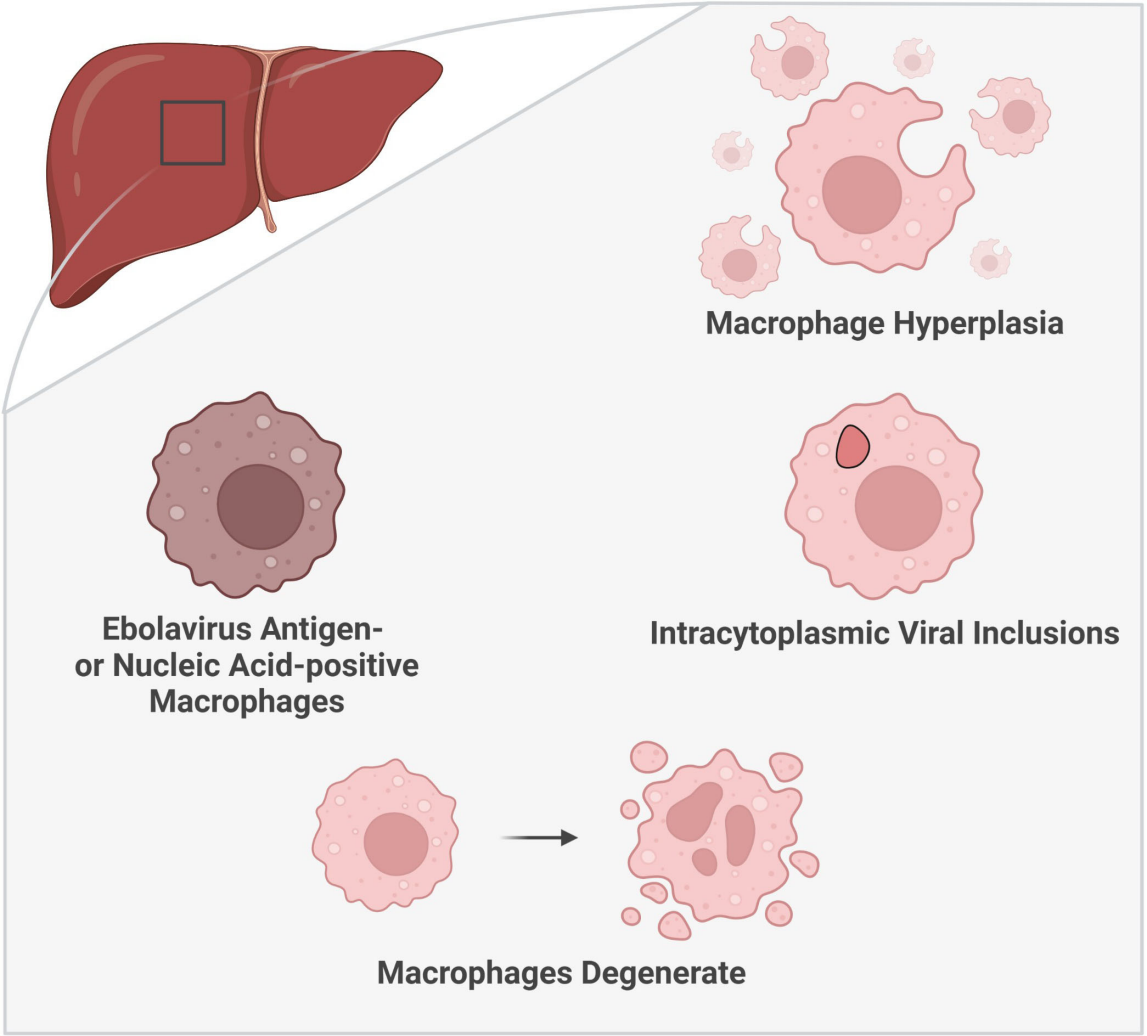
Histologic characteristics of macrophages in the liver during ebolavirus infection. The involvement of macrophages within the liver has been substantiated by multiple histologic findings: an increase in the number of macrophages (macrophage hyperplasia), the presence of viral inclusion bodies visible by light and electron microscopy, and the detection of virus antigen- or nucleic acid-positive macrophages. Moreover, macrophage degeneration has been observed later in the infection. (Created with Biorender.com).

Macrophage positivity for virus material is commonly reported in the liver in human autopsies and animal necropsies (rhesus macaques, chimpanzee (natural infection), mice, guinea pigs) (**Figure 3**) (Jaax et al., 1996; Bray et al., 1998; Connolly et al., 1999; Wyers et al., 1999; Bray, 2001; Raymond et al., 2011; Groseth et al., 2012; Hoenen et al., 2015; Spengler et al., 2016; Lavender et al., 2018; Woolsey et al., 2021). These cells are among the earliest cells positive for virus material in the liver. Ebolavirus RNA-positivity has been reported in Kupffer cells and monocytes as early as 2 days post-infection (DPI), with virus antigen-positive Kupffer cells detected at 3 DPI, in EBOV-infected cynomolgus macaques (Geisbert et al., 2003a). Kupffer cell virus antigen- or RNA-positivity has been reported as early as 3 DPI in EBOV-challenged rhesus macaques, as well as in mouse-adapted EBOV-challenged BALB/c mice (antigen+, RNA+); these cells were also observed at 1 DPI in guinea pigs (Gibb et al., 2001; Cross et al., 2015; Liu et al., 2022). Another study reported infected macrophages (non-Kupffer cells) at 2 DPI in green monkeys by electron microscopy, one day prior to the observation of infected Kupffer cells (Ryabchikova et al., 1999). In rhesus macaques, antigen-positive Kupffer cells are observed in both aerosol and oral challenge models (Johnson et al., 1995; Mire et al., 2016). Kupffer cell virus antigen-positivity can also vary based on the ebolavirus challenge species within the same animal model. In IFN-α/βR^−/−^ mice, virus antigen-positive Kupffer cells were noted in animals challenged with SUDV and EBOV, but not BDBV (Brannan et al., 2015). Similarly, in NSG™-SGM3 human immune system mice, virus antigen-positive Kupffer cells were more common following challenge with EBOV than RESTV (Spengler et al., 2018). Additionally, virus antigen accumulation in hepatic macrophages may correlate with the onset of symptomatic disease in macaques. For example, antigen-positive macrophage increases in a cynomolgus macaque model of EBOV infection occurred between 3-4 DPI, in association with the onset of fever, rash, anorexia, and dehydration (Geisbert et al., 2003a).

Ebolavirus infection in humans is associated with Kupffer cell hyperplasia (Zaki and Goldsmith, 1999) (**Figure 3**). Macrophages have also been reported to accumulate within the liver in primates (naturally infected with RESTV or TAFV or laboratory-challenged with BDBV) as well as in NSG™-SGM3 human immune system mice (laboratory-challenged with EBOV/Makona) (Geisbert et al., 1992; Wyers et al., 1999; Spengler et al., 2016; Woolsey et al., 2021). Macrophages also accumulate in areas of infection in macaques naturally infected with RESTV (Dalgard et al., 1992). Guinea pigs and NSG™-SGM3 human immune system mice present with a unique form of pathology, which can include the presence of granulomatous or granuloma-like foci containing macrophages (Ryabchikova et al., 1996; Mateo et al., 2011; Spengler et al., 2016). The source of this increase in macrophages within the liver ought to be explored as these cells can derive from multiple sources, including resident (Kupffer cell) or systemic sources (monocyte differentiation) (Guillot and Tacke, 2019).

The observation of ebolavirus inclusions within hepatic macrophages demonstrates that macrophages not only pick up viral material, as indicated by virus antigen-positive macrophages, but that they can also be directly infected by ebolaviruses. Hepatic macrophages have been shown to harbor intracytoplasmic ebolavirus inclusions in humans, rhesus macaques (light microscopy only), chimpanzees (natural infection, light microscopy only), mice, and guinea pigs (Ryabchikova et al., 1996; Goldsmith et al., 1997; Bray et al., 1998; Wyers et al., 1999; Gibb et al., 2001; Liu et al., 2022) (**Figure 3**). In humans, inclusion-containing Kupffer cells are often periportal (Goldsmith et al., 1997). In rhesus macaques, virions are observed to bud from Kupffer cells (Jaax et al., 1996). Kupffer cell degeneration or necrosis can be associated with Kupffer cells bearing virus inclusions in rhesus (light microscopy only) and cynomolgus macaques infected with EBOV, as well as in guinea pigs (Connolly et al., 1999; Geisbert et al., 2003a; Liu et al., 2022). In humans, plasma membrane proliferations may be observed in infected Kupffer cells (Goldsmith et al., 1997). Non-viral cytoplasmic tubuloreticular inclusions, observed in RESTV-infected cynomolgus macaques (naturally infected), may be distinguished from viral inclusions by electron microscopy and virus antigen staining. These inclusions are associated with the rough endoplasmic reticulum and have been reported in multiple cells, including circulating monocytes and macrophages as well as Kupffer cells (Geisbert et al., 1992). As such, care should be taken to distinguish viral inclusion bodies from non-viral inclusion bodies in pathological descriptions of these cells.

Moreover, histologic descriptions of macrophages within the liver during EVD abound, including notation of a shift toward macrophage degeneration as the disease progresses (**Figure 3**). Enlarged macrophages and hypertrophic Kupffer cells were reported in macaques naturally infected with RESTV and BDBV-challenged macaques, respectively (Geisbert et al., 1992; Woolsey et al., 2021). Macrophages containing Periodic Acid-Schiff-positive material have been documented in human EVD patients (World Health Organization/International Study Team, 1978). In a huNSG-A2 mouse model (NOD.Cg-*Prkdc^scid^* *Il2rg^tm1Wjl^* Tg(HLA-A2.1)1Enge/SzJ mice, severely immune-compromised, expressing HLA-A2.1, irradiated and engrafted with human CD34^+^ human hematopoietic stem cells) of EBOV and RESTV, virus replication correlated with inflammatory cell infiltration into the liver, including monocytes/macrophages, in mice succumbing to infection. In mice surviving RESTV infection, phagocytosis of RESTV antigen-positive cells by Iba1^+^ monocytes/macrophages was observed (Escudero-Pérez et al., 2019). Macrophages containing brown pigment, as well as macrophages with or without iron pigment, have also been reported (Dietrich et al., 1978; World Health Organization/International Study Team, 1978; Spengler et al., 2016; Escaffre et al., 2021). Additionally, fibrin deposition has been documented around hepatic macrophages positive for virus material, including infected macrophages, suggesting a potential role for macrophages in activation of the coagulation cascade (Geisbert et al., 2003a; Geisbert et al., 2003b). As the infection progresses, macrophage degeneration and necrosis occur, described in cynomolgus macaque and BALB/c mouse models of EVD (Gibb et al., 2001; Geisbert et al., 2003a). Degenerate or necrotic macrophages have also been reported in the liver of human patients as well as in RESTV-infected cynomolgus macaques (naturally infected) (Murphy, 1978; Geisbert et al., 1992). Kupffer cell necrosis was more frequent than macrophage necrosis in RESTV-infected cynomolgus macaques (natural infection) (Geisbert et al., 1992). Free ebolavirus virions have also been found in association with Kupffer cell debris (Connolly et al., 1999; Geisbert et al., 2003a).

### Spleen

Macrophage infection and association with viral material, phagocytic activity, and other findings support an active role of macrophages within the spleen during ebolavirus infection. Virus antigen-positive red pulp macrophages have been reported in human EVD cases (Martines et al., 2015). Ebolavirus antigen-positive macrophages or macrophage-like cells have also been reported in the spleen, including in either the white pulp or red pulp, across multiple animal models (macaques, mice, chimpanzees (natural infection), guinea pigs) and virus species (EBOV, SUDV, TAFV, BDBV) (Johnson et al., 1995; Jaax et al., 1996; Connolly et al., 1999; Wyers et al., 1999; Bray, 2001; Brannan et al., 2015; Herbert et al., 2015; Wong et al., 2016a; Woolsey et al., 2021). Macrophages within the spleen become positive for viral nucleic acid by Day 2 in EBOV-infected cynomolgus macaques and mouse-adapted EBOV-infected mice (Gibb et al., 2001; Geisbert et al., 2003a). Ebolavirus inclusions have also been reported in splenic macrophages in naturally infected macaques and a naturally infected chimpanzee (light microscopy only) as well as in ebolavirus-challenged macaques, guinea pigs, and mice (Geisbert et al., 1992; Jaax et al., 1996; Ryabchikova et al., 1996; Connolly et al., 1999; Wyers et al., 1999; Gibb et al., 2001). Compared to wild-type mice, fewer virus inclusions were observed in splenic macrophages in importin-α7^-/-^ mice (Gabriel et al., 2015). Non-viral inclusions were observed in circulating monocytes and macrophages in macaques naturally infected with RESTV (Geisbert et al., 1992). Tingible body macrophages were observed in rhesus macaque, guinea pig, and A129 interferon α/β receptor-deficient mouse models (Lever et al., 2012; Twenhafel et al., 2013; Twenhafel et al., 2015; Cross et al., 2015; Woolsey et al., 2021). Splenic macrophages containing necrotic cell debris were also reported in a mouse model of SUDV (Escaffre et al., 2021). Fibrin was reported in association with activated marginal zone and red pulp macrophages in EBOV-challenged macaques or near degenerate macrophages in a guinea pig model of EBOV (guinea pig-adapted virus) (Connolly et al., 1999; Geisbert et al., 2003b). TUNEL^+^ apoptotic bodies were observed in splenic macrophages as early as 2 DPI (Geisbert et al., 2003a). Ebolavirus infected macrophages were also noted to be degenerate or necrotic in a mouse-adapted EBOV mouse model (Gibb et al., 2001). Brown pigments have been observed in splenic macrophages in mouse models (Spengler et al., 2016; Escaffre et al., 2021). In Escaffre et al. (2021), these brown pigments were suspected to be due to hemosiderin or bilirubin from internal hemorrhage. Multinucleated giant cells have also been reported in a mouse model (Spengler et al., 2016). Future work exploring the potential transmission of virions from local infected macrophages to T cells could identify a contribution of macrophages to lymphocyte death, which is noted to occur in the spleen, as abortive T cell infection with ebolavirus has been suggested as a potential cause of T cell death (Younan et al., 2019; Jacob et al., 2020). Macrophages may also play a role in the clean-up of dead lymphocytes or other debris during infection, as evidenced by the presence of tingible body macrophages.

### Lung

**Figure 4:**
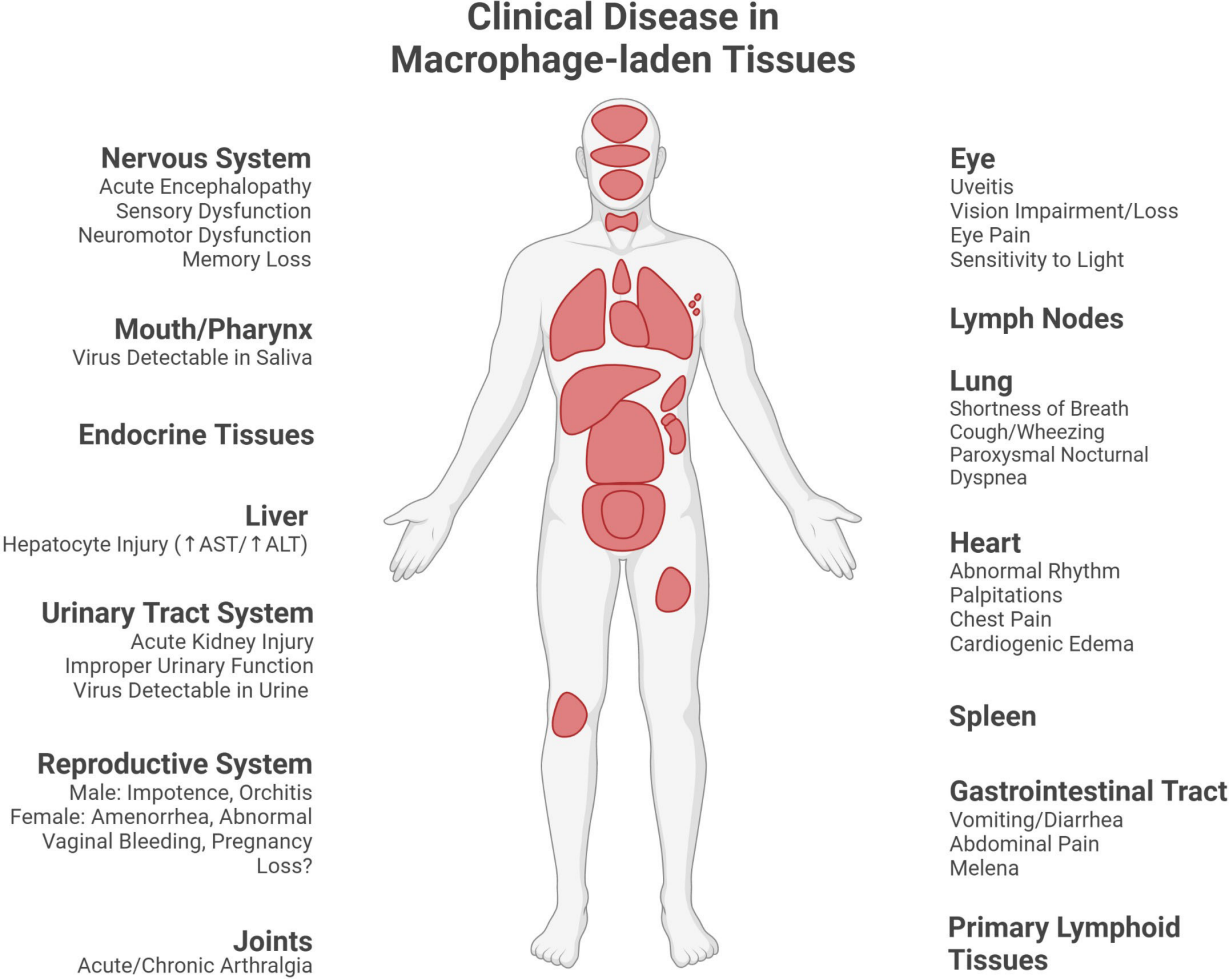
Clinical disease in macrophage-laden tissues during or following Ebola virus disease. Many of the observable manifestations of acute Ebola virus disease, as well as sequelae, are linked to the injury of organs containing macrophage populations (clinical signs and symptoms reported in the macrophage-laden tissues described in this paper as reviewed in Jacob et al. (2020)). (Created with Biorender.com).

Macrophage populations in the lungs are important to consider in EVD as pulmonary disease is present in both acute infection as well as in some recovered patients. Pulmonary symptoms can persist long after the resolution of acute EVD, with patients complaining of shortness of breath, cough, excess sputum, wheezing, and paroxysmal nocturnal dyspnea (Jacob et al., 2020) (**Figure 4**). Ebolavirus inclusion-containing, virus antigen-positive, or virus nucleic acid-positive alveolar macrophages, or virions sometimes in association with macrophages, have been reported in the lungs of human EVD patients (Goldsmith et al., 1997; Zaki and Goldsmith, 1999; Martines et al., 2015). Non-viral inclusions were observed in monocytes and circulating macrophages in macaques naturally infected with RESTV (Geisbert et al., 1992). Virus antigen-positive macrophages have been reported in the alveoli, alveolar interstitium, alveolar septae, tracheo-bronchial lymph nodes, or bronchial- or bronchiolar-associated lymphoid tissue in primate models of EVD (including aerosol challenge) or in primates naturally infected with RESTV or TAFV (Geisbert et al., 1992; Johnson et al., 1995; Jaax et al., 1996; Wyers et al., 1999; Twenhafel et al., 2013; Woolsey et al., 2021). Virus antigen-positive regions of macrophages and fibrin have been observed in the blood vessels of the nares, larynx, trachea, and bronchial tree in EBOV-challenged rhesus macaques (Jaax et al., 1996). In porcine models, virus antigen-positive macrophages have been reported in the lung, including alveolar and septal macrophages (Kobinger et al., 2011; Marsh et al., 2011; Weingartl et al., 2012; Nfon et al., 2013). In guinea pig models (including aerosol challenge), virus antigen- or nucleic acid-positive alveolar macrophages have been reported (Connolly et al., 1999; Steele et al., 2001; Cross et al., 2015; Twenhafel et al., 2015). Macrophages recently recruited from the circulation (Mac387^+^) that are also virus antigen-positive are present in the lungs of a porcine model of ebolavirus infection (Zwadlo et al., 1988; Goebeler et al., 1994; Nfon et al., 2013). Increased Mac387^+^ cells are also seen in the lungs of RESTV-infected cynomolgus macaques (natural infection) (Ikegami et al., 2002). Macrophage accumulations have been reported in the alveoli as well as in the alveolar walls in rhesus macaque models (including aerosol challenge) (Johnson et al., 1995; Jaax et al., 1996; Wong et al., 2016b). There were necrotic macrophages in the lymph nodes and lymphoid follicles of the respiratory tract in a green monkey model of EBOV (Ryabchikova et al., 1999). Inclusions have been reported in macrophages in the alveoli, vascular spaces, tracheo-bronchial lymph nodes, or interstitium of the lung in macaques naturally infected or challenged (including aerosol challenge) with ebolaviruses (Dalgard et al., 1992; Geisbert et al., 1992; Johnson et al., 1995; Jaax et al., 1996; Twenhafel et al., 2013). Inclusions have also been reported in alveolar macrophages in a RESTV porcine model and in an EBOV guinea pig model (aerosol challenge, light microscopy only) (Twenhafel et al., 2015; Haddock et al., 2021).

### Heart

While the cardiovascular system is strained during acute EVD and long-term cardiac sequelae can persist in patients, the inconsistency of macrophage association with virus material in this organ suggests other causes for cardiac injury. Severe EVD often includes the development of hypovolemic shock secondary to low blood volume and/or septic shock, which can include vascular leakage. Moreover, EVD survivors can continue to experience cardiac symptoms, including an irregular heart rate, heart murmurs, palpitations, and cardiogenic edema (Jacob et al., 2020) (**Figure 4**). While macrophages are present within the tissues of the heart, they are not consistently associated with the virus in this organ. In a patient who died from EBOV infection, CD163^+^ cardiac macrophages were not noted to be EBOV antigen-positive, though some cardiomyocytes did stain positive for the virus (McElroy et al., 2019). Similarly, no virus antigen-positive macrophages were observed in the heart of a wild chimpanzee that succumbed to natural TAFV infection (Wyers et al., 1999). In contrast, macrophages infiltrating heart valve tissue in EBOV-challenged Hartley guinea pigs were observed to contain intracytoplasmic inclusions suggestive of direct infection (light microscopy only) (Cooper et al., 2018). This inconsistency in the viral burden of cardiac macrophages sets these cells apart from macrophages routinely infected in other tissues, such as the liver and spleen. Elucidation of the difference between cardiac macrophages, or their tissue context, and those of more heavily burdened organs may shed light on macrophage susceptibility to ebolavirus infection as well as the role of macrophages in cardiac pathogenesis secondary to EVD.

### Joints

Macrophages may also play a role in the development of arthralgia during and after EVD as infected macrophages are present in these tissues. Patients can experience joint pain within the first few days of disease or as part of post-Ebola syndrome (Jacob et al., 2020) (**Figure 4**). Evidence for macrophage involvement within joints has been reported in a rhesus macaque challenge study. Some rhesus macaques challenged with mouse-adapted EBOV/Yambuku-Mayinga showed mild recruitment of macrophages (some of which showed signs of degeneration) into the stifle joint (knee). Intimal CD68^+^ macrophages in the stifle joint stained positive for EBOV GP antigen. Intimal and subintimal macrophages in the stifle synovium contained, or were near, virus particles, nucleocapsid inclusions, and budding virions. In contrast, few stifle joints from rhesus macaques challenged with EBOV/Kikwit or EBOV/Makona-C05 showed histologic pathologies despite the presence of virus antigen-positivity within the joint (Cooper et al., 2020). As specific descriptions of, or staining for, macrophages were not mentioned for the EBOV/Kikwit nor EBOV/Makona-C05 groups, comparison of macrophage-specific findings in joint tissue across multiple EBOV species would clarify how broadly the above findings apply. Nevertheless, the recruitment of macrophages into the joint and the detection of infected macrophages in this tissue supports their potential role in joint pain experienced by patients with EVD.

### Primary lymphoid tissues

Macrophage involvement within primary lymphoid tissues, including the thymus and bone marrow, may contribute to disruption of other immune cell lineages. Virus antigen-positive, as well as virus inclusion- and budding virion-bearing, macrophages were observed in the bone marrow of rhesus macaques challenged with EBOV. Bone marrow necrosis was observed in all macaques, along with hemorrhage in some cases (Jaax et al., 1996). Infected macrophages have also been reported in the bone marrow of guinea pigs challenged with EBOV (determination by light or electron microscopy not specifically stated) (Connolly et al., 1999). In the thymus, small foci of EBOV-positive macrophage-like cells were observed in cynomolgus macaques (determination by light or electron microscopy not specifically stated) (Geisbert et al., 2003a). Virus antigen-positive subcapsular macrophages were also observed in the thymus at 4 DPI in BALB/c mice challenged with mouse-adapted EBOV. Macrophage viral antigen-positivity preceded lymphocytolysis by a day, providing a temporal correlation for the role of macrophage infection in lymphocyte demise (Gibb et al., 2001). Whether the activation of infected or virus material-associated macrophages within these tissues impair their being a source of immune cells, including lymphocytes, should be further studied. In addition, the temporal correlation between macrophage virus antigen-positivity and lymphocytolysis shown by Gibb et al. (2001) suggests a potential cause-and-effect relationship that should be clarified. Given that infected macrophages could serve as a local tissue source of virus, this could lead to abortive T cell infection, which has been suggested to lead to T cell death (Younan et al., 2019).

### Lymph nodes

Macrophage histological descriptions and association with ebolavirus material within the lymph nodes has been reported across multiple animal models. Virus antigen- or nucleic acid-positive macrophages have been reported in lymph nodes in primate (including naturally infected) and guinea pig models of ebolaviruses, including in the subcapsular sinus, medullary sinus, or cortical sinus (Geisbert et al., 1992; Connolly et al., 1999; Wyers et al., 1999; Geisbert et al., 2003a). Virus antigen- or nucleic acid-positive macrophages are reported as early as 2-3 DPI in lymph nodes in a macaque model and 2 DPI in a mouse model (Gibb et al., 2001; Geisbert et al., 2003a). Virus inclusions have been reported in various lymph nodes across naturally infected macaques as well as ebolavirus-challenged macaques, guinea pigs, and mice (Geisbert et al., 1992; Jaax et al., 1996; Connolly et al., 1999; Gibb et al., 2001). Moreover, tingible body macrophages were observed in lymph nodes, except for in the axillary lymph nodes, in a rhesus macaque model of EBOV (Alves et al., 2016). In addition, fibrin-enclosed ebolavirus-infected macrophages were also reported in the inguinal lymph nodes in EBOV-infected macaques (Geisbert et al., 2003b). Lymph node sinus histiocytosis as well as macrophages and necrotic cell debris in the subcapsular and medullary sinuses were reported in an EBOV-challenged rhesus macaque model (Jaax et al., 1996). Increased virus antigen- or nucleic acid-positive macrophage counts in the axillary lymph nodes (at 48 hours post-infection) as well as macrophage degeneration were observed in guinea pigs (Ryabchikova et al., 1996; Connolly et al., 1999). The activity of macrophages within various lymph nodes and their association with virus material, or their direct infection by ebolaviruses, could promote lymphocyte depletion. Infected macrophages could serve as a local source of virus, which could promote abortive infection of T cells, which has been suggested as a potential cause of T cell death (Younan et al., 2019). Additionally, the presence of tingible body macrophages indicates that these cells may play a beneficial role by clearing debris accumulating during infection, which could serve as material for antigen presentation.

### Mouth and pharynx

Macrophage infection and pathological findings have also been reported in and around the mouth, one of the potential routes for ebolavirus transmission. EBOV has been isolated from saliva and transmission through ebolavirus exposure *via* the oral mucosa is possible (Jacob et al., 2020) (**Figure 4**). Degenerate macrophages, as well as RESTV antigen-positive macrophages in the submucosa and tonsillar lymphoid crypts, have been observed in the pharynx of RESTV-infected (natural infection) cynomolgus macaques (Geisbert et al., 1992). Macrophages positive for virus material have been observed in the *lamina propria* of the tongue, the olfactory mucosa, dental pulp, and the gingival submucosa in Strain 13 guinea pigs infected with guinea pig-adapted EBOV/Mayinga (Connolly et al., 1999). Histiocytic inflammation around foci of necrotic acinar cells in the parotid gland, which foci were virus antigen- or nucleic acid-positive in some cases, as well as a few macrophages with intracytoplasmic inclusions in salivary glands, were observed in outbred Hartley guinea pigs challenged with EBOV (light microscopy only) (Cooper et al., 2018). The response of macrophages to tissue injury as well as their infected status in these regions suggest the potential for macrophages to contribute to both the infection response in these tissues and virus transmission *via* the oral route.

### Gastrointestinal tract

Macrophage involvement in various portions of the intestinal tract and the pancreas suggest a role for macrophages in the gastrointestinal disease that is part of EVD. Patients with EVD commonly experience vomiting and diarrhea, which can lead to extensive fluid and solute losses (Jacob et al., 2020) (**Figure 4**). Ebolavirus-infected, inclusion-bearing, or virus antigen- or nucleic acid-positive monocytes or macrophages have been reported in intestinal tissues in a macaque model of EBOV (*lamina propria* and submucosa) and macaques naturally infected with RESTV as well as in the gut-associated lymphoid tissue in a mouse EBOV model and in the intestines and pancreas of guinea pigs (Cooper et al. (2018) only performed light microscopy) (Geisbert et al., 1992; Jaax et al., 1996; Connolly et al., 1999; Gibb et al., 2001; Cooper et al., 2018). Moreover, virus nucleic acid-positive monocytes/macrophages were observed in the vicinity of submucosal hemorrhages in the colon of EBOV/Kikwit-infected rhesus macaques. EBOV GP associated with CD68^+^ monocytes/macrophages was observed within the colonic mucosa (Reisler et al., 2018). In addition, in a TAFV-infected chimpanzee (natural infection), infiltrating macrophages were present in pyogranulomatous lesions in the intestine (Wyers et al., 1999). There were necrotic macrophages in the lymph nodes and lymphoid follicles of the gastrointestinal tract in a green monkey model of EBOV (Ryabchikova et al., 1999). Taken together, monocytes or macrophages, including cells infected by ebolaviruses or associated with ebolavirus material, are present within gastrointestinal tissues, and, in some cases, also observed near pathological intestinal lesions. Future study clarifying the macrophage phenotypes present in these tissues, as well as their mechanistic contribution to pathological lesion development/resolution would enrich the understanding of gastrointestinal injury during ebolavirus infection.

### Urinary tract

Macrophages are also present in the urinary tract during ebolavirus infection. Clinically, acute kidney injury can occur during ebolavirus infection and some survivors experience urinary tract dysfunction (**Figure 4**) (Jacob et al., 2020). In cynomolgus macaques, EBOV antigen-positive macrophage-like cells have been reported in the kidney (Geisbert et al., 2003a). However, in a TAFV-infected chimpanzee (natural infection), no antigen-positive macrophages were observed in the interstitium of the renal parenchyma (Wyers et al., 1999). Virus antigen- or nucleic acid-positive macrophages have also been reported in the kidney and bladder in a guinea pig model (Connolly et al., 1999). Viral inclusions have been reported in renal circulating and interstitial macrophages and circulating monocytes in RESTV-infected cynomolgus macaques (natural infection) (Geisbert et al., 1992). Enlarged macrophages present in the renal proximal tubules were observed in a RESTV-infected cynomolgus macaque (natural infection) (Ikegami et al., 2002). The presence of macrophages positive for viral material, or even containing viral inclusions, suggests their potential role in the infection within these tissues. Future work focused on the urinary tract could determine the possible beneficial or detrimental contributions of macrophages during the course of infection, including with respect to clinical findings, such as the development of acute kidney injury and chronic urinary tract dysfunction.

### Endocrine tissues

Virus antigen- and nucleic acid-positive, as well as infected, macrophages have also been observed in select endocrine tissues within the body. Virus antigen- or nucleic acid-positive or infected macrophages have been reported in the adrenal glands of macaques naturally infected with RESTV as well as macaque (light microscopy only) or mouse models of EBOV (Geisbert et al., 1992; Gibb et al., 2001; Geisbert et al., 2003a; Liu et al., 2022). Low numbers of inclusion-bearing macrophages have also been reported in the thyroid in a guinea pig model of EBOV. Virus antigen- or nucleic acid-positive as well as degenerate macrophages have also been reported within the thyroid in this model (light microscopy only) (Cooper et al., 2018). Taken together, these data demonstrate the presence of infected macrophages or macrophages associated with virus material in the adrenal gland and the thyroid. Future work should be done to clarify the mechanistic role of these cells, whether protective or detrimental, in the development or resolution of the lesions that can be present in these tissues during ebolavirus infection (Cooper et al., 2018; Liu et al., 2022).

### Nervous system

Macrophage involvement in the nervous system is important to consider. During acute disease, patients can experience encephalopathy (Jacob et al., 2020) (**Figure 4**). EBOV antigen-positive CD68^+^ microglia have been reported in the brain in a rhesus macaque surviving challenge (36 DPI) (Zeng et al., 2017). Moreover, EBOV antigen-positive CD68^+^ macrophages have been detected within the dorsal root ganglion (no EBOV antigen co-localization with microglia (Iba1^+^) or satellite cells (GFAP^+^)) in an EBOV-infected rhesus macaque. CD68^+^ macrophages were also increased in numbers in the interstitium of dorsal root ganglia and autonomic ganglia in EBOV-challenged rhesus macaques compared with controls. The CD68^+^ cells around dorsal root ganglia neurons were large, plump/ameboid, and had foamy cytoplasm (Liu et al., 2020). Inclusion bodies have been reported in macrophages within ganglia in EBOV-infected guinea pigs (light microscopy only) (Cooper et al., 2018). Inclusion bodies and filamentous EBOV particles were present in macrophages near the neuronal perikaryon in the ganglia of the seminal vesicle and adrenal gland in an EBOV-infected rhesus macaque model (Liu et al., 2020). Lipid-laden macrophages in an axillary/brachial nerve without inclusion bodies were also observed in an EBOV-infected guinea pig model (light microscopy only) (Cooper et al., 2018). Taken together, these findings demonstrate that ebolavirus-infected macrophages can be present within peripheral nervous tissues. Further work is warranted to determine whether the virus antigen-positive macrophages observed in the brain also harbor functional virions. Moreover, determining whether these cells contribute to clinical manifestations of neurologic disease during and following acute infection would add functional outcomes to these observations.

### Eye

**Figure 5:**
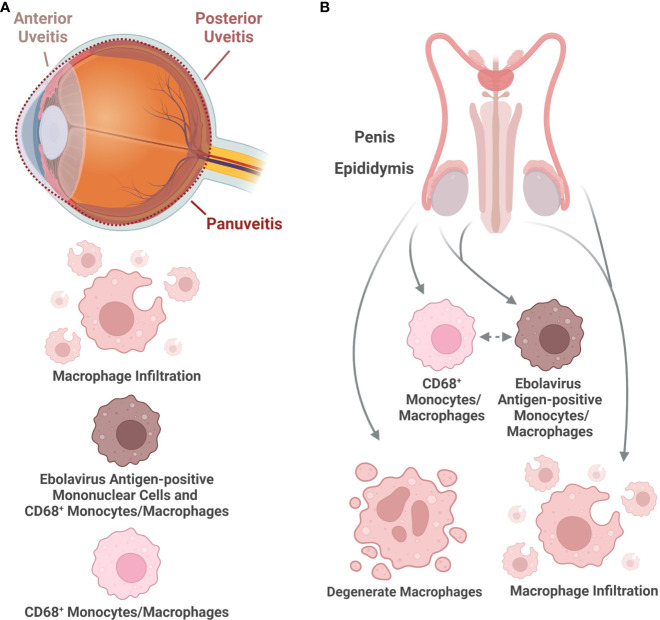
Histologic characteristics of macrophages in organs associated with long-term virus presence following ebolavirus infection. **(A)** The eye has been identified as a site harboring ebolaviruses well after the resolution of acute infection in certain cases. Inflammation of the eye can occur in the anterior or posterior chamber of the eye, as well as throughout the eye. Macrophage infiltration as well as macrophages positive for viral antigen have been observed in the eye, including in CD68+ macrophages. **(B)** The male reproductive tract has been associated with rare ebolavirus transmission. The following findings have been reported in macaques: Macrophages positive for viral antigen have been observed in the epididymis and penis. CD68+ macrophages, some of which are virus antigen-positive, have been observed in the epididymis. Macrophage infiltration during infection has also been observed in the epididymis and penis. Degenerate macrophages have been observed in the epididymis. (Created with Biorender.com).

Macrophages within the eye may contribute to virus persistence and ocular sequelae in EVD survivors. Patients can develop eye pain, vision alterations, and uveitis following recovery from infection (**Figure 4**). Three forms of uveitis have been reported: anterior uveitis, affecting the iris, ciliary body and anterior chamber of the eye, posterior uveitis, affecting the choroid or posterior retina, or panuveitis (**Figure 5A**) (Rosenbaum and Dick, 2018; Jacob et al., 2020). Macrophages were found to be among the cells infiltrating the conjunctival subepithelial tissue in a rhesus macaque which developed unilateral eye swelling following challenge with EBOV/Kikwit. Virus antigen-positive mononuclear cells, potentially of histiocytic origin, were present. Perivascular mononuclear cells associated with the choroid plexus were also occasionally virus antigen positive (Alves et al., 2016). Moreover, macrophages were among the cells infiltrating the ciliary body, iris, the sclera near the iridocorneal angles, as well as along cyclitic membranes in survivor macaques. In addition, inflammatory cells with macrophage morphology were present in the vitreous humor next to the retinal inner limiting membrane. CD68^+^ monocytes/macrophages were present in various parts of the eye and, in some cases, co-localized with EBOV antigen (Zeng et al., 2017). These studies demonstrate that infiltrating and virus antigen-positive macrophages are present in the tissues affected in EVD-related uveitis (**Figure 5A**). Further elucidation of macrophage-related mechanisms of ebolavirus persistence and inflammation within the eye, along with therapeutic strategies, may help prevent eye injury in convalescent patients. In addition, determination of whether virus antigen-positive macrophages also contain functional virions would clarify the extent to which macrophages may harbor functional virus within the eye following the resolution of acute infection.

### Reproductive system

Macrophages may also be important for sexual transmission and dysfunction by survivors of EVD. Sexual dysfunction in both men and women, including both orchitis and amenorrhea, as well as rare sexual transmission by men have been reported in survivors of EVD (Jacob et al., 2020) (**Figure 4**). In male reproductive tissues, macrophages have been reported in the interstitial connective tissue of the epididymis in a rhesus macaque surviving EVD. CD68^+^ monocytes/macrophages are among the cells within the epididymis, some of which were EBOV antigen positive, on Day 43 post-challenge. Necrotic cell debris within the epididymal tubular lumen included degenerate macrophages (Zeng et al., 2017). EBOV antigen-positive macrophages have also been reported in the epididymal interstitium and in the *lamina propria* of the penis in another rhesus macaque model of EBOV. In addition, EBOV antigen-positive circulating monocytes were also present in the *lamina propria* of the penis in this model. Macrophage infiltrates into the penile hypodermis and the submucosa of the urethra following EBOV infection have also been reported in rhesus macaques (Liu et al., 2022) (**Figure 5B**). In female reproductive tissues, SUDV antigen-positive cells included macrophages that were also positive for malarial parasite pigment within the placenta in a human case. Malarial parasite pigment-laden macrophages were also found in areas of fibrin deposition. In a human BDBV case, virus antigen-positive circulating macrophages were reported in the placenta, along with atypical macrophages, which had degenerate-appearing nuclei, cytoplasmic blebs, and potential viral inclusions (no virions were observed in the tissue by transmission electron microscopy) (Muehlenbachs et al., 2017). EBOV antigen- or nucleic acid-positive macrophages have also been reported within the endocervix, the ectocervix, the lamina propria or submucosa of the vagina, and the ovaries in a rhesus macaque model of EBOV (Liu et al., 2022). Virus inclusions within ovarian stromal macrophages have also been reported in a rhesus macaque model (Jaax et al., 1996). EBOV-infected guinea pigs can also have virus inclusion-bearing, virus antigen-positive, or virus nucleic acid-positive macrophages within the female genital tract (light microscopy only) (Cooper et al., 2018). The presence of macrophages positive for viral material or macrophages containing viral inclusions may make these cells a virus source for sexual or vertical transmission or indicate that they are contributors to the response to the infection in these tissues, which could modulate the development of acute or chronic reproductive tissue injury caused by EVD. Future examination of these various reproductive tissues by electron microscopy will help determine whether ebolavirus-infected macrophages are present.

## Discussion

Macrophages have long been recognized for their role in ebolavirus infection, with contributions to host susceptibility, the inflammatory response, and tissue injury. Ebolavirus entry into macrophages is enhanced by a M2-like phenotype and is facilitated by various host molecules, including multiple scavenger receptors. Both macrophage exposure to, and infection by, ebolaviruses result in the production of numerous inflammatory cytokines. *In vivo*, ebolavirus antigen- or nucleic acid-positive, as well as infected, macrophages have been observed in a multitude of tissues, from principal sites of pathological findings, like the liver and spleen, to tissues that are potential reservoir sites, including the eyes and testes.

**Figure 6:**
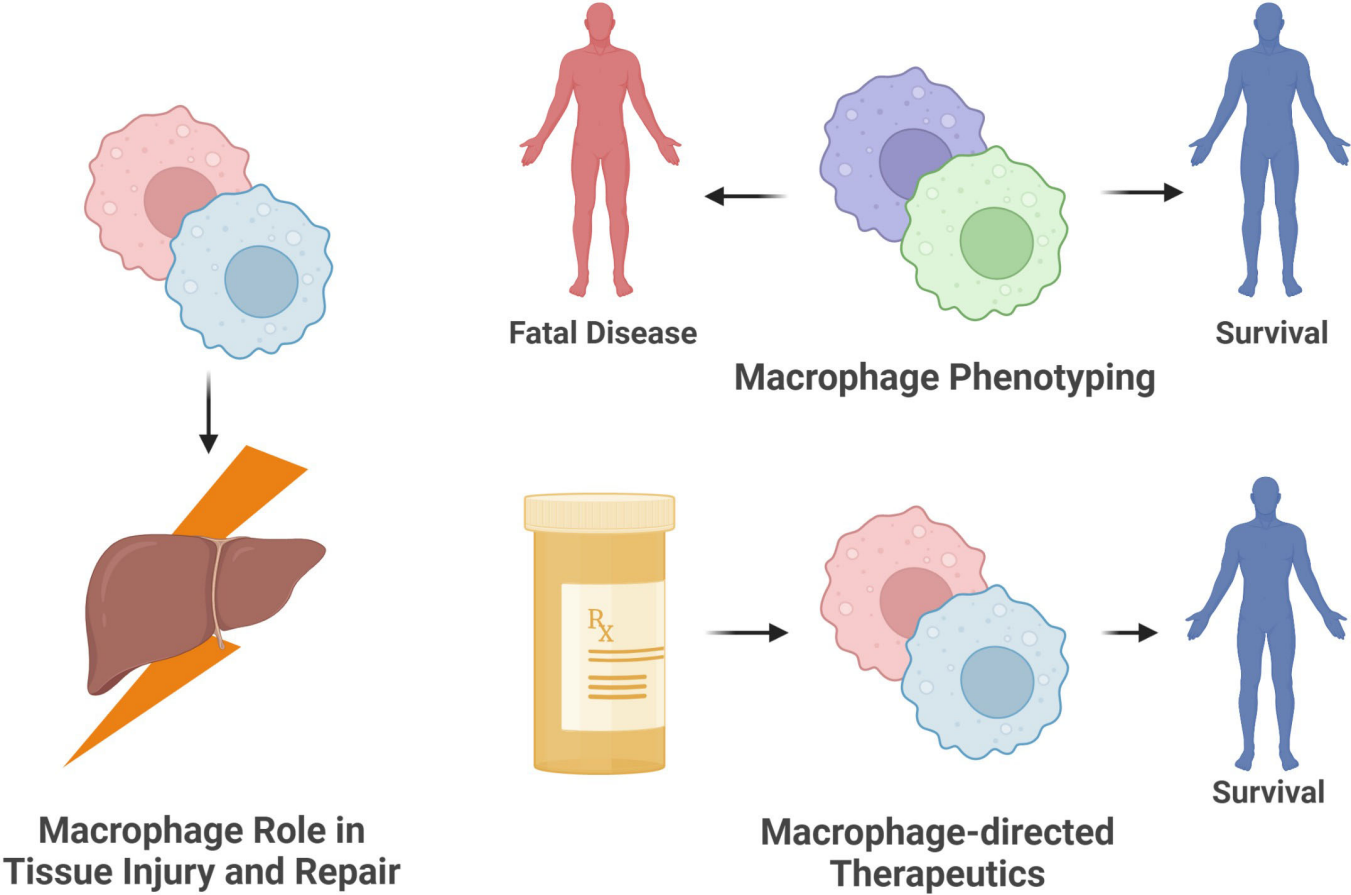
Opportunities for future research on the role of macrophages in ebolavirus infection. The ebolavirus field will benefit from (1) identifying the contribution of specific macrophage populations to fatal versus non-fatal infection, (2) defining the role of macrophages in the injury or repair of tissues during and following infection, and (3) promoting patient survival by developing macrophage-directed interventions suppressing harmful functions or enhancing beneficial functions of these cells. (Created with Biorender.com).

The mechanisms by which macrophages contribute to tissue injury *in vivo*, the macrophage phenotypes associated with disease severity, and macrophage-directed treatments all stand as opportunities to advance the understanding of, and countermeasures against, EVD (**Figure 6**). While pathological lesions are present in multiple organs, often in concert with the presence of macrophages, the extent of damaging versus reparative functions of macrophages remains to be determined. Moreover, identifying macrophage phenotypes contributing to disease versus healing using more recent and sophisticated phenotyping methods for macrophage populations, such as in the liver, will help clarify which macrophage populations are key contributors to severe disease outcomes (Guillot and Tacke, 2019). Identifying these cell populations and key contributory cellular pathways will not only expand understanding of the host response to ebolavirus infection but will also lay the foundation for enhanced therapeutic approaches countering EVD.

## Author contributions

TGW prepared the original draft and figures and implemented revisions thereof. DEM, OAS, JM, TS, RAR, ST, and EA provided critical feedback on draft versions of this work. MEC provided critical feedback on, and assisted with revisions of, the figures accompanying the manuscript. HLS and SP provided critical feedback on draft versions of this work as well as supervision, administration, resources, and funding of this work.

## Funding

This work was conducted with the support of the Institute for Translational Sciences at the University of Texas Medical Branch, supported in part by a Clinical and Translational Science Award NRSA (TL1) Training Core (TL1TR001440) from the National Center for Advancing Translational Sciences, National Institutes of Health (TW); Moody Endowment Award, Galveston, TX (2014-07 and LIME 19016) (HLS); John S. Dunn Foundation (SP).

## Acknowledgments

The authors would like to thank J. Trumble (Moody Medical Library, UTMB), A. DeVries (Moody Medical Library, UTMB) and A. Howard (Moody Medical Library, UTMB) for their help with literature search methods, reference acquisition, and reference formatting, the Department of Pathology (UTMB), and the Department of Microbiology and Immunology (UTMB).

## Conflict of interest

The authors declare that the research was conducted in the absence of any commercial or financial relationships that could be construed as a potential conflict of interest.

## Publisher’s note

All claims expressed in this article are solely those of the authors and do not necessarily represent those of their affiliated organizations, or those of the publisher, the editors and the reviewers. Any product that may be evaluated in this article, or claim that may be made by its manufacturer, is not guaranteed or endorsed by the publisher.

## References

[B1] Albariño C. G. Guerrero L. W. Jenks H. M. Chakrabarti A. K. Ksiazek T. G. Rollin P. E.. (2017). Insights into reston virus spillovers and adaption from virus whole genome sequences. PloS One 12, e0178224. doi: 10.1371/journal.pone.0178224 28542463 PMC5444788

[B2] Alves D. A. Honko A. N. Kortepeter M. G. Sun M. Johnson J. C. Lugo-Roman L. A.. (2016). Necrotizing scleritis, conjunctivitis, and other pathologic findings in the left eye and brain of an Ebola virus-infected rhesus macaque (Macaca mulatta) with apparent recovery and a delayed time of death. J. Infect. Dis. 213, 57–60. doi: 10.1093/infdis/jiv357 26153408 PMC4707191

[B3] Ayithan N. Bradfute S. B. Anthony S. M. Stuthman K. S. Dye J. M. Bavari S.. (2014). Ebola Virus-like particles stimulate type I interferons and proinflammatory cytokine expression through the toll-like receptor and interferon signaling pathways. J. Interferon Cytokine Res. 34, 79–89. doi: 10.1089/jir.2013.0035 24102579 PMC3924795

[B4] Baseler L. Chertow D. S. Johnson K. M. Feldmann H. Morens D. M. (2017). The pathogenesis of Ebola virus disease. Annu. Rev. Pathology: Mech. Dis. 12, 387–418. doi: 10.1146/annurev-pathol-052016-100506 27959626

[B5] Brannan J. M. Froude J. W. Prugar L. I. Bakken R. R. Zak S. E. Daye S. P.. (2015). Interferon α/β receptor-deficient mice as a model for Ebola virus disease. J. Infect. Dis. 212 Suppl 2, S282–S294. doi: 10.1093/infdis/jiv215 25943199

[B6] Bray M. (2001). The role of the type I interferon response in the resistance of mice to filovirus infection. J. Gen. Virol. 82, 1365–1373. doi: 10.1099/0022-1317-82-6-1365 11369881

[B7] Bray M. Davis K. Geisbert T. Schmaljohn C. Huggins J. (1998). A mouse model for evaluation of prophylaxis and therapy of Ebola hemorrhagic fever. J. Infect. Dis. 178, 651–661. doi: 10.1086/515386 9728532

[B8] Casals C. García-Fojeda B. Minutti C. M. (2019). Soluble defense collagens: Sweeping up immune threats. Mol. Immunol. 112, 291–304. doi: 10.1016/j.molimm.2019.06.007 31228661

[B9] Centers for Disease Control and Prevention (2022) History of Ebola outbreaks. Available at: https://www.cdc.gov/vhf/ebola/history/chronology.html.

[B10] Connolly B. M. Steele K. E. Davis K. J. Geisbert T. W. Kell W. M. Jaax N. K.. (1999). Pathogenesis of experimental Ebola virus infection in guinea pigs. J. Infect. Dis. 179 Suppl 1, S203–S217. doi: 10.1086/514305 9988186

[B11] Cooper T. K. Huzella L. Johnson J. C. Rojas O. Yellayi S. Sun M. G.. (2018). Histology, immunohistochemistry, and in situ hybridization reveal overlooked Ebola virus target tissues in the Ebola virus disease guinea pig model. Sci. Rep. 8, 1250. doi: 10.1038/s41598-018-19638-x 29352230 PMC5775334

[B12] Cooper T. K. Logue J. Liu D. X. Perry D. L. Hart R. J. Hischak A. M. W.. (2020). Filoviruses infect rhesus macaque synoviocytes in vivo and primary human synoviocytes in vitro. Am. J. Pathol. 190, 1867–1880. doi: 10.1016/j.ajpath.2020.05.013 32479821 PMC7456742

[B13] Cross R. W. Fenton K. A. Geisbert J. B. Mire C. E. Geisbert T. W. (2015). Modeling the disease course of Zaire ebolavirus infection in the outbred Guinea pig. J. Infect. Dis. 212 Suppl 2, S305–S315. doi: 10.1093/infdis/jiv237 26038397

[B14] Dahlmann F. Biedenkopf N. Babler A. Jahnen-Dechent W. Karsten C. B. Gnirß K.. (2015). Analysis of Ebola virus entry into macrophages. J. Infect. Dis. 212 Suppl 2, S247–S257. doi: 10.1093/infdis/jiv140 25877552 PMC4564540

[B15] Dalgard D. W. Hardy R. J. Pearson S. L. Pucak G. J. Quander R. Zack P. M.. (1992). Combined simian hemorrhagic fever and Ebola virus infection in cynomolgus monkeys. Lab. Anim. Sci. 42, 152–157. 1318446

[B16] Dietrich M. Schumacher H. H. Peters D. Knobloch J. (1978). “Human pathology of Ebola (Maridi) virus infection in the Sudan,” in Ebola Virus haemorrhagic fever (New York: Elsevier North-Holland Inc).

[B17] Domínguez-Soto A. Aragoneses-Fenoll L. Gómez-Aguado F. Corcuera M. T. Clária J. García-Monzón C.. (2009). The pathogen receptor liver and lymph node sinusoidal endotelial cell c-type lectin is expressed in human kupffer cells and regulated by PU.1. Hepatology 49, 287–296. doi: 10.1002/hep.22678 19111020 PMC7165556

[B18] Dominguez-Soto A. Aragoneses-Fenoll L. Martin-Gayo E. Martinez-Prats L. Colmenares M. Naranjo-Gomez M.. (2007). The DC-SIGN-related lectin LSECtin mediates antigen capture and pathogen binding by human myeloid cells. Blood 109, 5337–5345. doi: 10.1182/blood-2006-09-048058 17339424

[B19] Dutta M. Robertson S. J. Okumura A. Scott D. P. Chang J. Weiss J. M.. (2017). A systems approach reveals MAVS signaling in myeloid cells as critical for resistance to Ebola virus in murine models of infection. Cell Rep. 18, 816–829. doi: 10.1016/j.celrep.2016.12.069 28099857 PMC5289750

[B20] Escaffre O. Juelich T. L. Neef N. Massey S. Smith J. Brasel T.. (2021). STAT-1 knockout mice as a model for wild-type Sudan virus (SUDV). Viruses 13, 1388. doi: 10.3390/v13071388 34372594 PMC8310124

[B21] Escudero-Pérez B. Ruibal P. Rottstegge M. Lüdtke A. Port J. R. Hartmann K.. (2019). Comparative pathogenesis of Ebola virus and reston virus infection in humanized mice. JCI Insight 4, e126070. doi: 10.1172/jci.insight.126070 PMC6948759 31550241

[B22] Favier A. L. Gout E. Reynard O. Ferraris O. Kleman J. P. Volchkov V.. (2016). Enhancement of Ebola virus infection *via* ficolin-1 interaction with the mucin domain of GP glycoprotein. J. Virol. 90, 5256–5269. doi: 10.1128/JVI.00232-16 26984723 PMC4934759

[B23] Gabriel G. Feldmann F. Reimer R. Thiele S. Fischer M. Hartmann E.. (2015). Importin-α7 is involved in the formation of Ebola virus inclusion bodies but is not essential for pathogenicity in mice. J. Infect. Dis. 212 Suppl 2, S316–S321. doi: 10.1093/infdis/jiv240 26185094 PMC4564549

[B24] Geisbert T. W. Hensley L. E. Larsen T. Young H. A. Reed D. S. Geisbert J. B.. (2003a). Pathogenesis of Ebola hemorrhagic fever in cynomolgus macaques: evidence that dendritic cells are early and sustained targets of infection. Am. J. Pathol. 163, 2347–2370. doi: 10.1016/S0002-9440(10)63591-2 14633608 PMC1892369

[B25] Geisbert T. W. Jahrling P. B. Hanes M. A. Zack P. M. (1992). Association of Ebola-related reston virus particles and antigen with tissue lesions of monkeys imported to the united states. J. Comp. Pathol. 106, 137–152. doi: 10.1016/0021-9975(92)90043-t 1597531

[B26] Geisbert T. W. Young H. A. Jahrling P. B. Davis K. J. Kagan E. Hensley L. E. (2003b). Mechanisms underlying coagulation abnormalities in ebola hemorrhagic fever: overexpression of tissue factor in primate monocytes/macrophages is a key event. J. Infect. Dis. 188, 1618–1629. doi: 10.1086/379724 14639531

[B27] Gibb T. R. Bray M. Geisbert T. W. Steele K. E. Kell W. M. Davis K. J.. (2001). Pathogenesis of experimental Ebola Zaire virus infection in BALB/c mice. J. Comp. Pathol. 125, 233–242. doi: 10.1053/jcpa.2001.0502 11798240

[B28] Goebeler M. Roth J. Teigelkamp S. Song C. (1994). The monoclonal antibody MAC387 detects an epitope on the calcium-binding protein MRP14. J. Leukoc. Biol. 55, 259–261. doi: 10.1002/jlb.55.2.259 7507970

[B29] Goldsmith C. S. Rollin P. E. Zhang X. H. Peters C. J. Zaki S. R. (1997). Ebola Virus hemorrhagic fever, Zaire 1995: An ultrastructural study. Microscopy Microanalysis 3, 77–78. doi: 10.1017/S1431927600007273

[B30] Greenberg A. Huber B. R. Liu D. X. Logue J. P. Hischak A. M. W. Hart R. J.. (2020). Quantification of viral and host biomarkers in the liver of rhesus macaques: A longitudinal study of Zaire ebolavirus strain kikwit (EBOV/Kik). Am. J. Pathol. 190, 1449–1460. doi: 10.1016/j.ajpath.2020.03.003 32275904 PMC7322367

[B31] Groseth A. Marzi A. Hoenen T. Herwig A. Gardner D. Becker S.. (2012). The Ebola virus glycoprotein contributes to but is not sufficient for virulence *in vivo* . PloS Pathog. 8, e1002847. doi: 10.1371/journal.ppat.1002847 22876185 PMC3410889

[B32] Guillot A. Tacke F. (2019). Liver macrophages: Old dogmas and new insights. Hepatol. Commun. 3, 730–743. doi: 10.1002/hep4.1356 31168508 PMC6545867

[B33] Gupta M. Mahanty S. Ahmed R. Rollin P. E. (2001). Monocyte-derived human macrophages and peripheral blood mononuclear cells infected with ebola virus secrete MIP-1alpha and TNF-alpha and inhibit poly-IC-induced IFN-alpha in vitro. Virology 284, 20–25. doi: 10.1006/viro.2001.0836 11352664

[B34] Haddock E. Saturday G. Feldmann F. Hanley P. W. Okumura A. Lovaglio J.. (2021). Reston virus causes severe respiratory disease in young domestic pigs. Proc. Natl. Acad. Sci. U.S.A. 118, e2015657118. doi: 10.1073/pnas.2015657118 33443221 PMC7812766

[B35] Herbert A. S. Davidson C. Kuehne A. I. Bakken R. Braigen S. Z. Gunn K. E.. (2015). Niemann-pick C1 is essential for ebolavirus replication and pathogenesis in vivo. mBio 6, e00565–e00515. doi: 10.1128/mBio.00565-15 26015498 PMC4447246

[B36] Hintz K. A. Rassias A. J. Wardwell K. Moss M. L. Morganelli P. M. Pioli P. A.. (2002). Endotoxin induces rapid metalloproteinase-mediated shedding followed by up-regulation of the monocyte hemoglobin scavenger receptor CD163. J. Leukoc. Biol. 72, 711–717. doi: 10.1189/jlb.72.4.711 12377940

[B37] Hoenen T. Marzi A. Scott D. P. Feldmann F. Callison J. Safronetz D.. (2015). Soluble glycoprotein is not required for Ebola virus virulence in Guinea pigs. J. Infect. Dis. 212 Suppl 2, S242–S246. doi: 10.1093/infdis/jiv111 25957965 PMC4564536

[B38] Ikegami T. Miranda M. E. Calaor A. B. Manalo D. L. Miranda N. J. Niikura M.. (2002). Histopathology of natural Ebola virus subtype reston infection in cynomolgus macaques during the Philippine outbreak in 1996. Exp. Anim. 51, 447–455. doi: 10.1538/expanim.51.447 12451705

[B39] Jaax N. K. Davis K. J. Geisbert T. J. Vogel P. Jaax G. P. Topper M.. (1996). Lethal experimental infection of rhesus monkeys with Ebola-Zaire (Mayinga) virus by the oral and conjunctival route of exposure. Arch. Pathol. Lab. Med. 120, 140–155. 8712894

[B40] Jacob S. T. Crozier I. Fischer W. A. Hewlett A. Kraft C. S. Vega M. A.. (2020). Ebola Virus disease. Nat. Rev. Dis. Primers 6, 13. doi: 10.1038/s41572-020-0147-3 32080199 PMC7223853

[B41] Johnson E. Jaax N. White J. Jahrling P. (1995). Lethal experimental infections of rhesus monkeys by aerosolized Ebola virus. Int. J. Exp. Pathol. 76, 227–236. 7547435 PMC1997182

[B42] Judson S. Prescott J. Munster V. (2015). Understanding ebola virus transmission. Viruses 7, 511–521. doi: 10.3390/v7020511 25654239 PMC4353901

[B43] Kobinger G. P. Leung A. Neufeld J. Richardson J. S. Falzarano D. Smith G.. (2011). Replication, pathogenicity, shedding, and transmission of Zaire ebolavirus in pigs. J. Infect. Dis. 204, 200–208. doi: 10.1093/infdis/jir077 21571728

[B44] Kühl A. Pöhlmann S. (2012). How Ebola virus counters the interferon system. Zoonoses Public Health 59, 116–131. doi: 10.1111/j.1863-2378.2012.01454.x 22958256 PMC7165950

[B45] Kuhn J. H. Amarasinghe G. K. Perry D. L. (2021). “Filoviridae,” in Fields virology: Emerging viruses, vol. 1 . Eds. Howley P. M. Knipe D. M. (Philadelphia: Lippincott Williams & Wilkins), 449–503.

[B46] Lavender K. J. Williamson B. N. Saturday G. Martellaro C. Griffin A. Hasenkrug K. J.. (2018). Pathogenicity of Ebola and marburg viruses is associated with differential activation of the myeloid compartment in humanized triple knockout-bone marrow, liver, and thymus mice. J. Infect. Dis. 218, S409–S417. doi: 10.1093/infdis/jiy269 30085162 PMC6249575

[B47] Leung L. W. Martinez O. Reynard O. Volchkov V. E. Basler C. F. (2011). Ebola Virus failure to stimulate plasmacytoid dendritic cell interferon responses correlates with impaired cellular entry. J. Infect. Dis. 204, S973–S977. doi: 10.1093/infdis/jir331 21987778 PMC3189990

[B48] Lever M. S. Piercy T. J. Steward J. A. Eastaugh L. Smither S. J. Taylor C.. (2012). Lethality and pathogenesis of airborne infection with filoviruses in A129 α/β -/- interferon receptor-deficient mice. J. Med. Microbiol. 61, 8–15. doi: 10.1099/jmm.0.036210-0 21852521

[B49] Liu D. X. Cooper T. K. Perry D. L. Huzella L. M. Hischak A. M. W. Hart R. J.. (2022). Expanded histopathology and tropism of Ebola virus in the rhesus macaque model: Potential for sexual transmission, altered adrenomedullary hormone production, and early viral replication in liver. Am. J. Pathol. 192, 121–129. doi: 10.1016/j.ajpath.2021.09.009 34626576 PMC8759036

[B50] Liu D. X. Perry D. L. Cooper T. K. Huzella L. M. Hart R. J. Hischak A. M. W.. (2020). Peripheral neuronopathy associated with ebola virus infection in rhesus macaques: A possible cause of neurological signs and symptoms in human ebola patients. J. Infect. Dis. 222, 1745–1755. doi: 10.1093/infdis/jiaa304 PMC7751569 32498080

[B51] Møller H. J. (2012). Soluble CD163. Scand. J. Clin. Lab. Invest. 72, 1–13. doi: 10.3109/00365513.2011.626868 22060747

[B52] Mahanty S. Hutchinson K. Agarwal S. Mcrae M. Rollin P. E. Pulendran B. (2003). Cutting edge: Impairment of dendritic cells and adaptive immunity by Ebola and lassa viruses. J. Immunol. 170, 2797–2801. doi: 10.4049/jimmunol.170.6.2797 12626527

[B53] Malvy D. McElroy A. K. de Clerck H. Günther S. van Griensven J. (2019). Ebola Virus disease. Lancet 393, 936–948. doi: 10.1016/S0140-6736(18)33132-5 30777297

[B54] Marsh G. A. Haining J. Robinson R. Foord A. Yamada M. Barr J. A.. (2011). Ebola Reston virus infection of pigs: clinical significance and transmission potential. J. Infect. Dis. 204 Suppl 3, S804–S809. doi: 10.1093/infdis/jir300 21987755

[B55] Martines R. B. Ng D. L. Greer P. W. Rollin P. E. Zaki S. R. (2015). Tissue and cellular tropism, pathology and pathogenesis of Ebola and marburg viruses. J. Pathol. 235, 153–174. doi: 10.1002/path.4456 25297522

[B56] Martinez O. Johnson J. C. Honko A. Yen B. Shabman R. S. Hensley L. E.. (2013a). Ebola Virus exploits a monocyte differentiation program to promote its entry. J. Virol. 87, 3801–3814. doi: 10.1128/jvi.02695-12 PMC3624207 23345511

[B57] Martinez O. Ndungo E. Tantral L. Miller E. H. Leung L. W. Chandran K.. (2013b). A mutation in the Ebola virus envelope glycoprotein restricts viral entry in a host species- and cell-type-specific manner. J. Virol. 87, 3324–3334. doi: 10.1128/JVI.01598-12 23302883 PMC3592116

[B58] Mateo M. Carbonnelle C. Reynard O. Kolesnikova L. Nemirov K. Page A.. (2011). VP24 is a molecular determinant of Ebola virus virulence in guinea pigs. J. Infect. Dis. 204 Suppl 3, S1011–S1020. doi: 10.1093/infdis/jir338 21987737

[B59] McElroy A. K. Shrivastava-Ranjan P. Harmon J. R. Martines R. B. Silva-Flannery L. Flietstra T. D.. (2019). Macrophage activation marker soluble CD163 associated with fatal and severe Ebola virus disease in humans. Emerg. Infect. Dis. 25, 290–298. doi: 10.3201/eid2502.181326 30666927 PMC6346465

[B60] Mire C. E. Geisbert J. B. Agans K. N. Deer D. J. Fenton K. A. Geisbert T. W. (2016). Oral and conjunctival exposure of nonhuman primates to low doses of Ebola makona virus. J. Infect. Dis. 214, S263–S267. doi: 10.1093/infdis/jiw149 27284090 PMC5050459

[B61] Muehlenbachs A. de la Rosa Vázquez O. Bausch D. G. Schafer I. J. Paddock C. D. Nyakio J. P.. (2017). Ebola Virus disease in pregnancy: Clinical, histopathologic, and immunohistochemical findings. J. Infect. Dis. 215, 64–69. doi: 10.1093/infdis/jiw206 27226206

[B62] Murphy F. A. (1978). Pathology of Ebola virus infection. In: Ebola Virus haemorrhagic fever (New York: Elsevier North-Holland Inc). Available at: http://www.enivd.de/EBOLA/ebola-17.htm#TopOfPage (Accessed August 15, 2022).

[B63] Newton K. Dixit V. M. (2012). Signaling in innate immunity and inflammation. Cold Spring Harb. Perspect. Biol. 4, a006049. doi: 10.1101/cshperspect.a006049 22296764 PMC3282411

[B64] Nfon C. K. Leung A. Smith G. Embury-Hyatt C. Kobinger G. Weingartl H. M. (2013). Immunopathogenesis of severe acute respiratory disease in Zaire ebolavirus-infected pigs. PloS One 8, e61904. doi: 10.1371/journal.pone.0061904 23626748 PMC3633953

[B65] Olejnik J. Forero A. Deflubé L. R. Hume A. J. Manhart W. A. Nishida A.. (2017). Ebolaviruses associated with differential pathogenicity induce distinct host responses in human macrophages. J. Virol. 91, e00179-17. doi: 10.1128/jvi.00179-17 28331091 PMC5432886

[B66] Raymond J. Bradfute S. Bray M. (2011). Filovirus infection of STAT-1 knockout mice. J. Infect. Dis. 204 Suppl 3, S986–S990. doi: 10.1093/infdis/jir335 21987780

[B67] Reisler R. B. Zeng X. Schellhase C. W. Bearss J. J. Warren T. K. Trefry J. C.. (2018). Ebola Virus causes intestinal tract architectural disruption and bacterial invasion in non-human primates. Viruses 10, 513. doi: 10.3390/v10100513 PMC6213817 30241284

[B68] Rhein B. A. Powers L. S. Rogers K. Anantpadma M. Singh B. K. Sakurai Y.. (2015). Interferon-γ inhibits Ebola virus infection. PloS Pathog. 11, e1005263. doi: 10.1371/journal.ppat.1005263 26562011 PMC4643030

[B69] Rogers K. J. Brunton B. Mallinger L. Bohan D. Sevcik K. M. Chen J.. (2019). IL-4/IL-13 polarization of macrophages enhances Ebola virus glycoprotein-dependent infection. PloS Negl. Trop. Dis. 13, e0007819. doi: 10.1371/journal.pntd.0007819 31825972 PMC6905523

[B70] Rogers K. J. Maury W. (2018). The role of mononuclear phagocytes in Ebola virus infection. J. Leukoc. Biol. 104, 717–727. doi: 10.1002/JLB.4RI0518-183R 30095866

[B71] Rogers K. J. Shtanko O. Stunz L. L. Mallinger L. N. Arkee T. Schmidt M. E.. (2021). Frontline science: CD40 signaling restricts RNA virus replication in mϕs, leading to rapid innate immune control of acute virus infection. J. Leukoc. Biol. 109, 309–325. doi: 10.1002/JLB.4HI0420-285RR 32441445 PMC7774454

[B72] Rogers K. J. Shtanko O. Vijay R. Mallinger L. N. Joyner C. J. Galinski M. R.. (2020). Acute plasmodium infection promotes interferon-Gamma-Dependent resistance to Ebola virus infection. Cell Rep. 30, 4041–4051.e4. doi: 10.1016/j.celrep.2020.02.104 32209467 PMC7172281

[B73] Rosenbaum J. T. Dick A. D. (2018). The eyes have it: A rheumatologist’s view of uveitis. Arthritis Rheumatol. 70, 1533–1543. doi: 10.1002/art.40568 29790291 PMC6160350

[B74] Rougeron V. Feldmann H. Grard G. Becker S. Leroy E. M. (2015). Ebola And marburg haemorrhagic fever. J. Clin. Virol. 64, 111–119. doi: 10.1016/j.jcv.2015.01.014 25660265 PMC11080958

[B75] Ryabchikova E. I. Kolesnikova L. V. Luchko S. V. (1999). An analysis of features of pathogenesis in two animal models of Ebola virus infection. J. Infect. Dis. 179 Suppl 1, S199–S202. doi: 10.1086/514293 9988185

[B76] Ryabchikova E. Kolesnikova L. Smolina M. Tkachev V. Pereboeva L. Baranova S.. (1996). Ebola Virus infection in guinea pigs: presumable role of granulomatous inflammation in pathogenesis. Arch. Virol. 141, 909–921. doi: 10.1007/BF01718165 8678836

[B77] Schnittler H. J. Feldmann H. (1998). Marburg and Ebola hemorrhagic fevers: does the primary course of infection depend on the accessibility of organ-specific macrophages? Clin. Infect. Dis. 27, 404–406. doi: 10.1086/517704 9709901

[B78] Shapouri-Moghaddam A. Mohammadian S. Vazini H. Taghadosi M. Esmaeili S. A. Mardani F.. (2018). Macrophage plasticity, polarization, and function in health and disease. J. Cell Physiol. 233, 6425–6440. doi: 10.1002/jcp.26429 PMC13482560 29319160

[B79] Simmons G. Reeves J. D. Grogan C. C. Vandenberghe L. H. Baribaud F. Whitbeck J. C.. (2003). DC-SIGN and DC-SIGNR bind ebola glycoproteins and enhance infection of macrophages and endothelial cells. Virology 305, 115–123. doi: 10.1006/viro.2002.1730 12504546

[B80] Skytthe M. K. Graversen J. H. Moestrup S. K. (2020). Targeting of CD163+ Macrophages in Inflammatory and Malignant Diseases. Int. J. Mol. Sci. 21, 5497. doi: 10.3390/ijms21155497 PMC7432735 32752088

[B81] Soilleux E. J. Morris L. S. Leslie G. Chehimi J. Luo Q. Levroney E.. (2002). Constitutive and induced expression of DC-SIGN on dendritic cell and macrophage subpopulations in situ and in vitro. J. Leukoc. Biol. 71, 445–457. doi: 10.1189/jlb.71.3.445 11867682

[B82] Spengler J. R. Lavender K. J. Martellaro C. Carmody A. Kurth A. Keck J. G.. (2016). Ebola Virus replication and disease without immunopathology in mice expressing transgenes to support human myeloid and lymphoid cell engraftment. J. Infect. Dis. 214, S308–S318. doi: 10.1093/infdis/jiw248 27601621 PMC5050473

[B83] Spengler J. R. Saturday G. Lavender K. J. Martellaro C. Keck J. G. Nichol S. T.. (2018). Severity of disease in humanized mice infected with Ebola virus or reston virus is associated with magnitude of early viral replication in liver. J. Infect. Dis. 217, 58–63. doi: 10.1093/infdis/jix562 PMC5853858 29087482

[B84] Stantchev T. S. Zack-Taylor A. Mattson N. Strebel K. Broder C. C. Clouse K. A. (2019). Cytokine effects on the entry of filovirus envelope pseudotyped virus-like particles into primary human macrophages. Viruses 11, 889. doi: 10.3390/v11100889 PMC6832363 31547585

[B85] Steele K. Crise B. Kuehne A. Kell W. (2001). Ebola Virus glycoprotein demonstrates differential cellular localization in infected cell types of nonhuman primates and guinea pigs. Arch. Pathol. Lab. Med. 125, 625–630. doi: 10.1043/0003-9985(2001)1252.0.CO;2 11300932

[B86] Ströher U. West E. Bugany H. Klenk H. D. Schnittler H. J. Feldmann H. (2001). Infection and activation of monocytes by marburg and Ebola viruses. J. Virol. 75, 11025–11033. doi: 10.1128/JVI.75.22.11025-11033.2001 11602743 PMC114683

[B87] Twenhafel N. A. Mattix M. E. Johnson J. C. Robinson C. G. Pratt W. D. Cashman K. A.. (2013). Pathology of experimental aerosol Zaire ebolavirus infection in rhesus macaques. Vet. Pathol. 50, 514–529. doi: 10.1177/0300985812469636 23262834

[B88] Twenhafel N. A. Shaia C. I. Bunton T. E. Shamblin J. D. Wollen S. E. Pitt L. M.. (2015). Experimental aerosolized guinea pig-adapted Zaire ebolavirus (variant: Mayinga) causes lethal pneumonia in guinea pigs. Vet. Pathol. 52, 21–25. doi: 10.1177/0300985814535612 24829285

[B89] Versteeg K. Menicucci A. R. Woolsey C. Mire C. E. Geisbert J. B. Cross R. W.. (2017). Infection with the makona variant results in a delayed and distinct host immune response compared to previous Ebola virus variants. Sci. Rep. 7, 9730. doi: 10.1038/s41598-017-09963-y 28852031 PMC5574898

[B90] Wahl-Jensen V. Kurz S. Feldmann F. Buehler L. K. Kindrachuk J. DeFilippis V.. (2011). Ebola Virion attachment and entry into human macrophages profoundly effects early cellular gene expression. PloS Negl. Trop. Dis. 5, e1359. doi: 10.1371/journal.pntd.0001359 22028943 PMC3196478

[B91] Weingartl H. M. Embury-Hyatt C. Nfon C. Leung A. Smith G. Kobinger G. (2012). Transmission of Ebola virus from pigs to non-human primates. Sci. Rep. 2, 811. doi: 10.1038/srep00811 23155478 PMC3498927

[B92] Wong G. He S. Wei H. Kroeker A. Audet J. Leung A.. (2016a). Development and characterization of a Guinea pig-adapted Sudan virus. J. Virol. 90, 392–399. doi: 10.1128/JVI.02331-15 26491156 PMC4702555

[B93] Wong G. Qiu X. de la Vega M. A. Fernando L. Wei H. Bello A.. (2016b). Pathogenicity comparison between the kikwit and makona Ebola virus variants in rhesus macaques. J. Infect. Dis. 214, S281–S289. doi: 10.1093/infdis/jiw267 27651412 PMC5050479

[B94] Woolsey C. Borisevich V. Agans K. N. Fenton K. A. Cross R. W. Geisbert T. W. (2021). Bundibugyo ebolavirus survival is associated with early activation of adaptive immunity and reduced myeloid-derived suppressor cell signaling. mBio 12, e0151721. doi: 10.1128/mBio.01517-21 34372693 PMC8406165

[B95] WHO/International Study Team (1978). Ebola Haemorrhagic fever in Sudan 1976. Bull World Health Organ 56, 247–270. 307455 PMC2395561

[B96] World Health Organization (2022) Ebola Virus disease - democratic republic of the Congo. Available at: https://www.who.int/emergencies/disease-outbreak-news/item/2022-DON398.

[B97] Wyers M. Formenty P. Cherel Y. Guigand L. Fernandez B. Boesch C.. (1999). Histopathological and immunohistochemical studies of lesions associated with Ebola virus in a naturally infected chimpanzee. J. Infect. Dis. 179 Suppl 1, S54–S59. doi: 10.1086/514300 9988165

[B98] Younan P. Ramanathan P. Graber J. Gusovsky F. Bukreyeva A. (2017). The toll-like receptor 4 antagonist eritoran protects mice from lethal filovirus challenge. mBio 8, e00226-17. doi: 10.1128/mBio.00226-17 28442605 PMC5405229

[B99] Younan P. Santos R. I. Ramanathan P. Iampietro M. Nishida A. Dutta M.. (2019). Ebola Virus-mediated T-lymphocyte depletion is the result of an abortive infection. PloS Pathog. 15, e1008068. doi: 10.1371/journal.ppat.1008068 31648236 PMC6812753

[B100] Zaki S. R. Goldsmith C. S. (1999). Pathologic features of filovirus infections in humans. Curr. Top. Microbiol. Immunol. 235, 97–116. doi: 10.1007/978-3-642-59949-1_7 9893381

[B101] Zeng X. Blancett C. D. Koistinen K. A. Schellhase C. W. Bearss J. J. Radoshitzky S. R.. (2017). Identification and pathological characterization of persistent asymptomatic Ebola virus infection in rhesus monkeys. Nat. Microbiol. 2, 17113. doi: 10.1038/nmicrobiol.2017.113 28715405

[B102] Zhang F. Ren S. Zuo Y. (2014). DC-SIGN, DC-SIGNR and LSECtin: C-type lectins for infection. Int. Rev. Immunol. 33, 54–66. doi: 10.3109/08830185.2013.834897 24156700

[B103] Zwadlo G. Brüggen J. Gerhards G. Schlegel R. Sorg C. (1988). Two calcium-binding proteins associated with specific stages of myeloid cell differentiation are expressed by subsets of macrophages in inflammatory tissues. Clin. Exp. Immunol. 72, 510–515. 3048809 PMC1541584

